# TransCRISPR–sgRNA design tool for CRISPR/Cas9 experiments targeting specific sequence motifs

**DOI:** 10.1093/nar/gkad355

**Published:** 2023-05-09

**Authors:** Tomasz Woźniak, Weronika Sura, Marta Kazimierska, Marta Elżbieta Kasprzyk, Marta Podralska, Agnieszka Dzikiewicz-Krawczyk

**Affiliations:** Institute of Human Genetics, Polish Academy of Sciences, Poznań, Poland; Institute of Human Genetics, Polish Academy of Sciences, Poznań, Poland; Institute of Human Genetics, Polish Academy of Sciences, Poznań, Poland; Institute of Human Genetics, Polish Academy of Sciences, Poznań, Poland; Institute of Human Genetics, Polish Academy of Sciences, Poznań, Poland; Institute of Human Genetics, Polish Academy of Sciences, Poznań, Poland

## Abstract

Eukaryotic genomes contain several types of recurrent sequence motifs, e.g. transcription factor motifs, miRNA binding sites, repetitive elements. CRISPR/Cas9 can facilitate identification and study of crucial motifs. We present transCRISPR, the first online tool dedicated to search for sequence motifs in the user-provided genomic regions and design optimal sgRNAs targeting them. Users can obtain sgRNAs for chosen motifs, for up to tens of thousands of target regions in 30 genomes, either for the Cas9 or dCas9 system. TransCRISPR provides user-friendly tables and visualizations, summarizing features of identified motifs and designed sgRNAs such as genomic localization, quality scores, closest transcription start sites and others. Experimental validation of sgRNAs for MYC binding sites designed with transCRISPR confirmed efficient disruption of the targeted motifs and effect on expression of MYC-regulated genes. TransCRISPR is available from https://transcrispr.igcz.poznan.pl/transcrispr/.

## Graphical Abstract

**Graphical Abstract:**
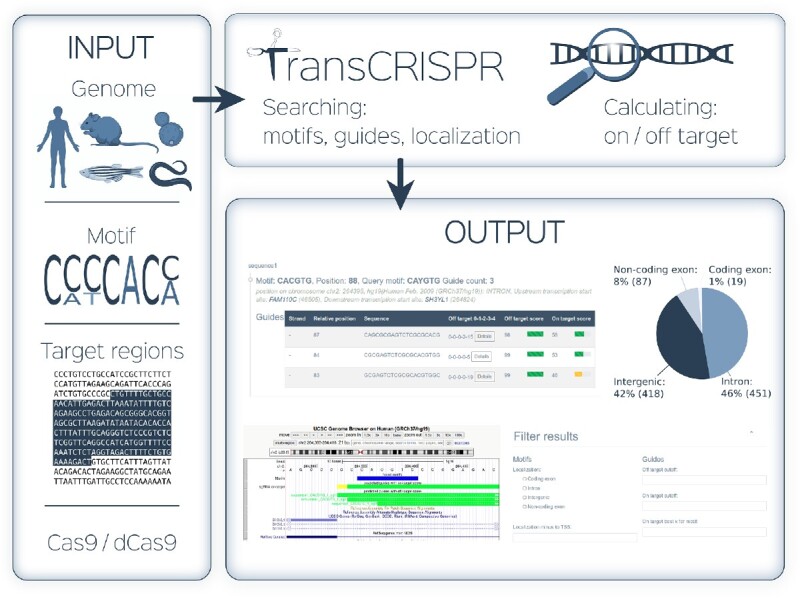
TransCRISPR searches for specific sequence motifs in the user-provided genomic regions and designs optimal sgRNAs targeting them. Results are shown in downloadable tables and visualizations, summarizing features of identified motifs and designed sgRNAs such as genomic localization, quality scores, closest transcription start sites and others.

## INTRODUCTION

Several types of recurrent sequence motifs exist in eukaryotic genomes and are important components of complex regulatory networks (1). For example, transcription factors (TFs) recognize specific sequence motifs in DNA and regulate target gene expression (2). MicroRNAs bind to their target transcripts via short complementary seed sequences (3). Splicing also depends on the recognition of specific sequences by the spliceosome (4). The role and significance of these motifs might be examined by their blocking or disruption, e.g. with use of CRISPR/Cas9 system (5–11). Clustered Regularly Interspaced Short Palindromic Repeats/Cas9 (CRISPR/Cas9) has become one of the most powerful tools for genome editing and revolutionized genome engineering. However, to perform reliable and informative CRISPR/Cas9 experiments, high specificity and efficiency of the approach are required. In answer to this need, numerous online tools have been designed. Several online CRISPR/Cas9 tools are available which allow designing the most optimal single-guide RNAs (sgRNAs) targeting specific sequences and predict their off- and on-target scores to increase their specificity and efficiency (12–16). Although they offer a wide range of possibilities, none of them allows searching for a specific motif in a given sequence and designing sgRNAs targeting this motif.

Here, we present a highly versatile online tool, transCRISPR, created to identify specific motifs in the sequence of interest and to design sgRNAs with optimal off- and on-target scores. It can be applied both for single sequences as well as for large lists of genome coordinates, enabling design of sgRNA libraries for genome-wide CRISPR/Cas9 screens.

## MATERIALS AND METHODS

### Data

Genomic coordinates for coding exons, non-coding exons, introns and transcription start sites (TSS) were downloaded from UCSC using a database interface. Full download command: mysql {genome_name} -h genome-mysql.soe.ucsc.edu -u genome -A -e ‘select * from ncbiRefSeqCurated’ -NB > {genome_file}. These data were further automatically processed to create specialized .bed files with genes and localization elements for each of available genomes. Whole genome sequences were downloaded as FASTA files.

### Implementation

TransCRISPR is created using Django (with Python programming language). MariaDB is used as a database, Celery with Redis as a query system, Daphne for websocket communication, Nginx as a web server, and Bootstrap-based Gentelella for a layout with Highcharts as a data visualization library. Docker with Docker Compose is used for management purposes. Off-targets are identified using the Cas-OFFinder (17) with a maximum of 4 (standard option) or 3 (rapid option) mismatches and later the CFD score is calculated for each off-target, as well as a cumulative CFD score (17, 18). For on-target value calculation a dockerized version of Azimuth is used (18). Two queue systems are available: for short calculations and for larger queries, so that short tasks can be proceeded quickly. Software is freely available online: https://transcrispr.igcz.poznan.pl.

Search of the motif positions is performed either with exact search in case of motifs defined as sequences with no IUPAC code or with regular expressions in case of IUPAC code in the sequence. In case of motif matrices, they are converted to IUPAC sequence using a selected rule set and then searched with regular expressions. Reverse complementary sequences are also generated and used for search on the reverse strand. For each of the found motif positions, potential guides are generated using rules for either Cas9 or dCas9.

In case of target sequences defined as coordinates, respective sequences are selected from downloaded genome files and extended by 30 nucleotides before and 30 nucleotides after defined coordinates. This enables design of guides for motifs at the border of the sequence. This approach is not possible in case of sequences defined as raw sequences or in FASTA format.

Localization of motifs in relation to genes is determined as follows. Firstly, data downloaded from UCSC are sorted and saved to special .bed files containing data from a single chromosome, sorted by given sequence start. For each chromosome respective .bed file is being sequentially searched for previously sorted motif positions. In case a motif is on a boundary (e.g. intron-exon, exon-intergenic etc.) or is localized in a position where different transcript variants differ with respect to intron/exon, the following hierarchy is applied: coding exon–non-coding exon–intron–intergenic.

For determination of the closest up- and downstream TSS, data from UCSC are similarly downloaded, sorted and saved to .bed files containing gene localization. During the analysis, the TSSs for the closest upstream and downstream gene for each of the sorted motifs are selected and saved.

For each motif and guide, a name is generated. If a target sequence name is given in the input (header in FASTA format or last column in .bed file) this name is used as prefix, in other cases a generic prefix is created.

## RESULTS

TransCRISPR is an online tool dedicated to designing sgRNAs targeting various sequence motifs. This software performs several steps to calculate and display results for given input: (i) processing sequences; (ii) processing motifs and finding motifs in sequences; (iii) calculating off-targets and on-targets; (iv) finding localization and calculating statistics.

### User interface

**Figure 1.**
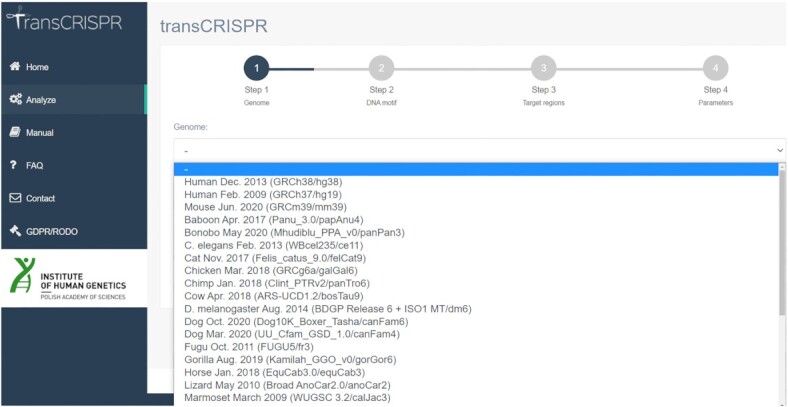
TransCRISPR interface and query steps.

To run a query, the user provides input data and selects available options (Figure 1). In Step 1, the reference genome is selected. Currently, thirty genome assemblies are available, including e.g. human, mouse, rat, fruit fly, zebrafish, *C. elegans* and others.

TransCRISPR offers several ways of submitting queries. In Step 2, sequence motifs might be entered directly in the window or uploaded as FASTA or comma separated file, or as a motif matrix in various formats, including output files of programs analyzing transcription factors (e.g. JASPAR, TRANSFAC). Motifs provided as a sequence can contain A, C, G, T nucleotides or IUPAC codes. Next, in Step 3 the target regions where motifs will be searched for are provided. Target sequence may be pasted directly in the window or uploaded as a text (FASTA or coma separated format) or as genomic coordinates.

**Figure 2.**
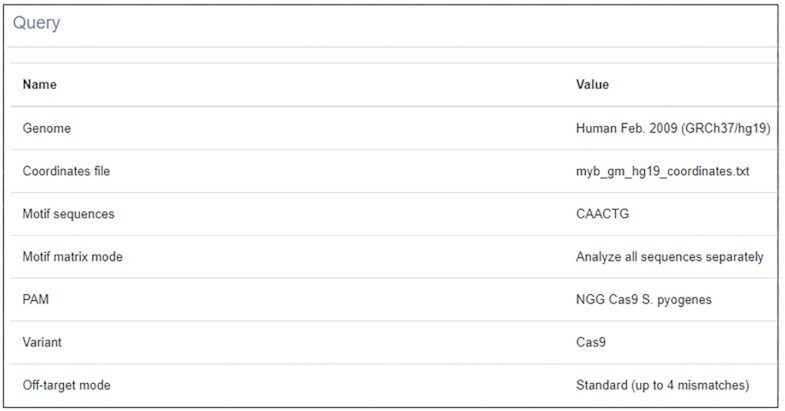
Summary of query details available on the result page.

In Step 4, several parameters are defined. If motifs were entered as a motif matrix, the user can choose criteria according to which motifs will be generated from the matrix (see Manual for detailed information). TransCRISPR currently supports the canonical *S. pyogenes* PAM NGG as well as variants: NGA and NGCG (19). Next, one can choose between the Cas9, dCas9 and custom variants which define how the sgRNAs are searched with respect to the motifs. In the Cas9 variant, only guides that lead to the cut within the motifs (taking into account that the cut occurs 3 nt upstream of PAM) are designed. In the dCas9 variant, any guides that overlap with at least one nucleotide of the motifs are included. The third option is ‘Custom’ where the user defines the maximum distance of PAM from the motif. This allows to search for PAM within a given range from the motif and design relevant sgRNAs. As a standard, for all found sgRNAs off-targets up to four mismatches are analyzed. To reduce the analysis time, the ‘rapid’ option can be chosen which includes only off-targets with up to three mismatches. The user can optionally enter their e-mail address to be informed when calculations are done. During the analysis, the user is informed about its progress and current step, as well as about the task queue. For convenience, the details of the query might be checked later (Figure 2). Results are available on the website for seven days.

### Analysis results and data visualization

The results page provides information about the number of found motifs and sgRNAs, an average number of guides per motif, and average on- and off-target scores, which are additionally presented on histograms. Distribution of guides per motif and their genomic localization are presented on downloadable pie charts. Information about found motifs includes: motif sequence, their position in the uploaded genomic sequence and localization in relevance to genes (coding or non-coding exon, intron or intergenic), and the genomic positions of transcription start sites (TSSs) for the closest up- and downstream gene. Information about the genomic localization of motifs and closest TSSs is available only if genomic coordinates for target regions were provided. Next, sgRNAs found for the motifs are presented in the table, where their sequence, relative position in the target region and DNA strand are shown, together with the calculated on- and off-target scores. It is possible to view the details of the most significant off-targets.

Results may be filtered by several parameters, which are described in detail in the manual. In some instances, identified motifs may overlap partially and in such a situation some sgRNAs may be duplicated in the motif view. To obtain the list of nonredundant sgRNAs, the user should switch to the ‘Unique guides’ mode.

The results can be downloaded in several formats (xslx, csv, tsv, bed). In the Excel file, separate sheets provide results per motifs or per unique guides. It is also possible to download the track to visualize motifs and guides together with their on- and off-target scores coded by colors in the UCSC Genome Browser. Moreover, by clicking Display in Genome Browser the user is directly taken to the Genome Browser with this track loaded.

A detailed explanation of preparing the query and analyzing results is provided in the manual available on the transCRISPR webpage, also as a downloadable pdf. To get familiar with transCRISPR and available options, it is advised to run one of the preloaded examples.

Summary of the features available in transCRISPR and comparison with other available tools for sgRNA design is presented in Table 1.

**Table 1.** Comparison of features offered by transCRISPR and other sgRNA design programs

	transCRISPR (this paper)	CRISPick (18,20)	CLD (21)	CRISPOR (12)	E-CRISP (13)	CHOPCHOP (22)	GuideScan2 (15)	CRISPRscan (16)
**GENERAL**								
Webserver	+	+	-	+	+	+	+	+
Command line interface	-	-	+	+	-	+	+	-
Batch design (multiple sites)	+	+	+	+	+	+ (in command line)	+	-
Targeting specific motifs	+	-	-	-	-	-	-	-
Multiple motifs	+	-	-	-	-	-	-	-
**INPUT**								
Motifs as sequence	+	-	-	-	-	-	-	-
Motifs as matrix (various formats)	+	-	-	-	-	-	-	-
Target as sequence	+	+	-	+	+	-	-	+
Target as coordinates	+	+	+	+	-	+	+	-
Target as gene name	-	+	+	+ (in batch mode)	+	+	+	+
Paired sgRNA design	-	-	+	-	+	+	+	-
Number of genome assemblies	30	6	31	806	55	375	7	22
Number of available PAM	3	4	custom PAM	38	17/custom PAM	7/custom PAM	2	5
**OUTPUT**								
Online results	+	-	-	+	+	+	+	+
Downloadable result	+	+	+	+	+	+	+	+
Downloadable track (eg. .bed, .gtf)	+	-	+	-	+	+	+	-
Redirection to genome browser	+	-	-	+	-	+	-	- (CRISPRscan predictions available for 9 species as tracks in the UCSC Genome Browser)
Embedded display	-	-		+	+	+	+	+
On-target value	Azimuth2.0 (16,18,23)	Appropriate algorithms depending on Cas enzyme	Azimuth 2.0 and SSC score (20); custom score allowed	Azimuth 2.0, CRISPRscan (16) and several others	Azimuth 2.0 and SSC score	Azimuth 2.0, CRISPRscan and several others	Azimuth 2.0	Azimuth 2.0 and CRISPRscan
Off-target value	CFDscore (18)	CFD score	Bowtie, Bowtie2 or Blast	MIT (24) and CFD score	Bowtie or Bowtie2 alignment with user-specified no. of tolerated mismatches	Bowtie alignment, sgRNAs are scored according to the no. of mismatches	Specificity score based on CFD	CFD score
Summary statistics	+	-	-	-	+	-	-	-
List of off-targets	+	-	-	+	-	+	+	Top 30
List of unique guides	+	-	-	-	-	-	-	-
Information about motif localization and closest genes	+	-	-	-	-	-	-	-
Primers for cloning/PCR/restriction enzymes	-	-	-	+	+	+	-	+
**FILTERING RESULTS**								
Localization (e.g.coding exon, non-coding exon, intron)	+	-	-	-	+ (at query)	+ (at query)	+ (only for exons)	-
X nucleotides from TSS	+	-	-	-	-	+ (at query)	-	-
On-target treshold	+	-	-	-	-	-	+ (at query)	-
Off-target treshold	+	-	-	-	+ (at query)	- (filtering by no. of mismatches)	+ (at query)	-
Removing motifs with no guides	+	-	-	-	-	-	-	-
Picking x best guides	+	+ (at query)	-	-	-	-	+ (at query)	-
Sorting data	by several features	-	-	by several features	-	by several features	by several features	-
**USAGE**								
Targeting transcription factor binding sites	+	-	-	-	-	-	-	-
Targeting repetitive elements	+	-	-	-	-	-	-	-
Targeting miRNA binding sites	+	-	-	-	-	-	-	-
CRISPRko	+	+	+	+	+	+	+	+
CRISPRi	+	+	+	-	+	+	-	-
CRISPRa	-	+	+	-	+	+	-	-
N-terminal tagging	-	-	+	-	+	-	-	-
C-terminal tagging	-	-	+	-	+	-	-	-
Gene targeting	-	+	+	+	+	+	+	+
Targeting non-coding regions	+	+ but sgRNAs in coding exons are favored	+	+	only exon flanking sequences	only UTRs and promoters	+	+

CRISPRko, CRISPR knockout; CRISPRi, CRISPR interference; CRISPRa, CRISPR activation.

### Case 1: example of library design

**Figure 3.**
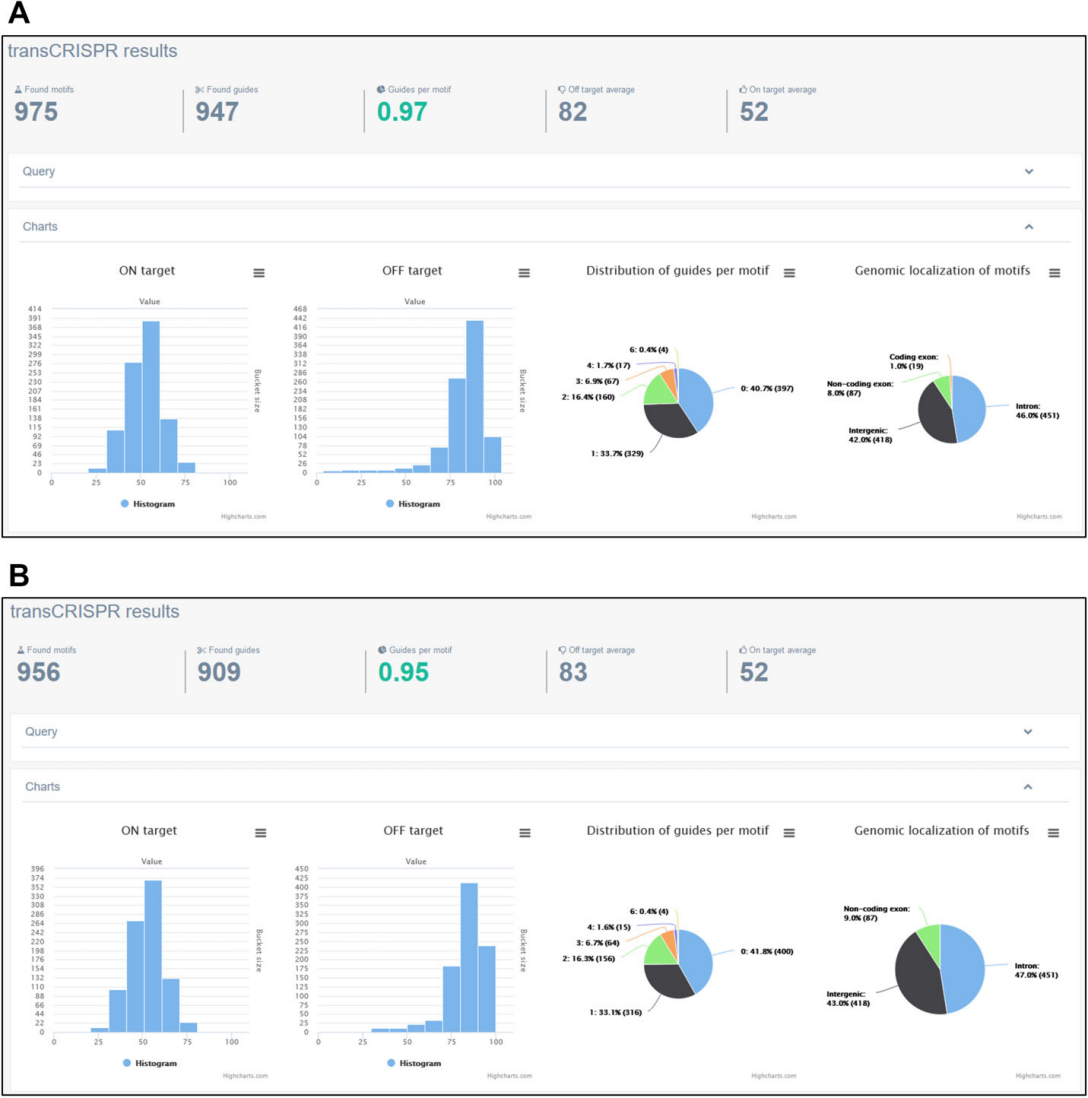
Statistics of transCRISPR results for the example query in Cas9 mode. The screenshots present initial results of analysis described in the example (**A**) and results after filtering by genomic localization of motifs and off-target scores (**B**).

**Figure 4.**
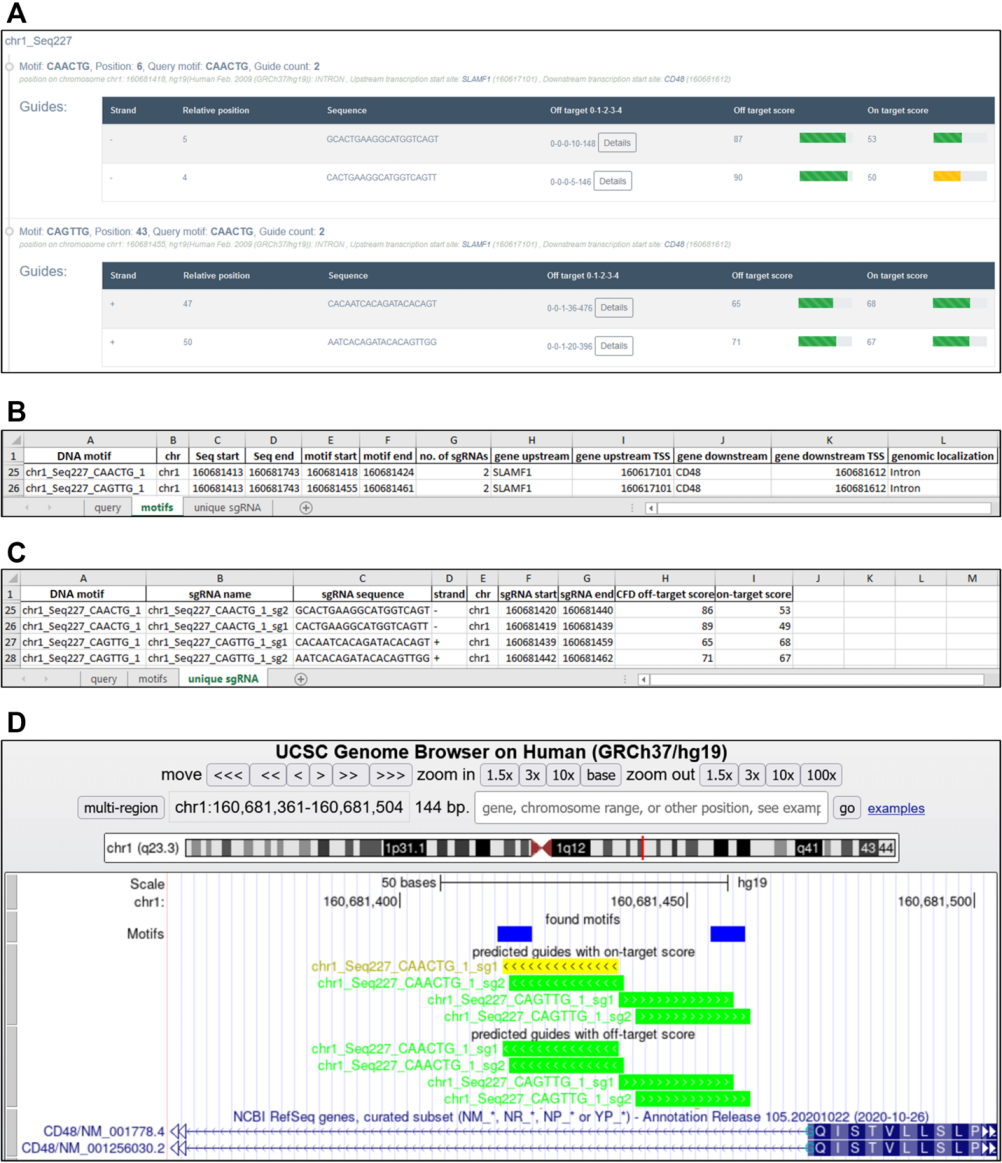
Different modes of viewing transCRISPR results. The screenshots present the same motifs and guides (**A**) directly on the results page; (**B**, **C**) in the downloaded xslx file (information on motifs – B, information on unique guides - C) and (**D**) as tracks in Genome Browser. Colors of sgRNAs bars depict on- and off-target values range.

We used transCRISPR to find motifs and sgRNAs within the ChIP peaks for the MYB transcription factor in human GM12878 cells (query details in Figure 2, 3748 MYB ChIP peaks retrieved from UCSC Table Browser). First, we chose NGG PAM, Cas9 variant and standard off-target mode (Figure 3). After the calculations are finished, the upper panel shows the summary of motifs and guides (Figure 3A). 59.3% of the identified 975 motifs were targeted by 947 guides (mostly 1 or 2 sgRNAs per motif; for some up to six sgRNAs were designed). The average on- and off-target scores were above 50 which indicated an overall good quality of designed sgRNAs. Detailed information provided on histograms showed that the majority of sgRNAs had off-target scores above 70, only a few below 50. The predicted cutting efficiency was medium as the majority of sgRNAs had the on-target score around 50. The identified motifs were mainly localized in the introns or non-coding exons, much less in intergenic regions and only a few in coding exons. Next, we filtered the results to exclude motifs present in coding regions and guides with off-target scores <30. As a result, we obtained 956 motifs and 909 guides with the average off-target score increased to 83 (Figure 3B). Figure 4 shows various modes of presenting results by transCRISPR.

**Figure 5.**
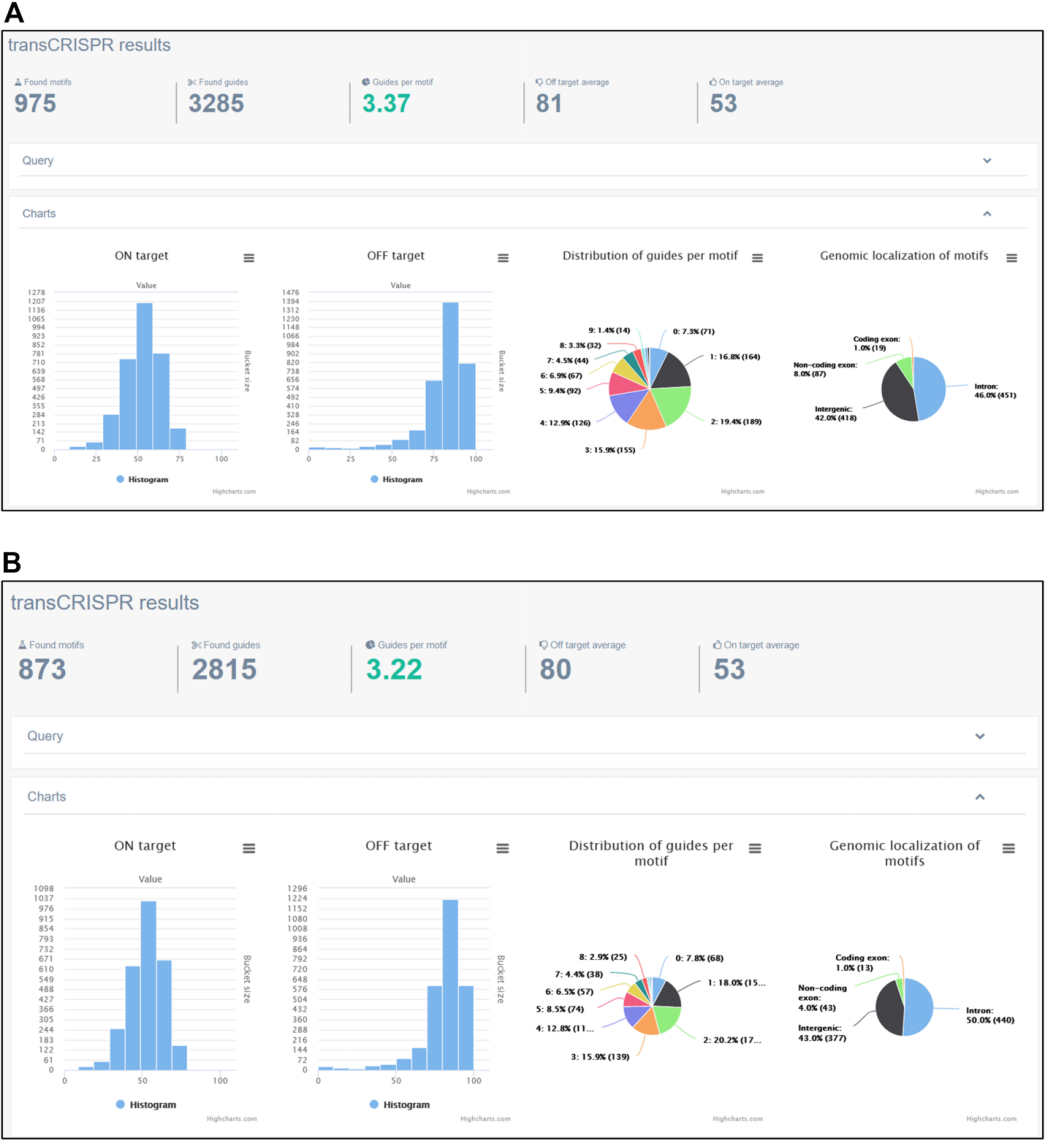
Statistics of transCRISPR results for the example query in dCas9 mode. The screenshots present initial results of analysis described in the example (**A**) and results after filtering by localization of motifs relative to TSS (**B**).

Changing the Cas9 variant in the query to dCas9 increased the number of motifs targeted by guides to >92% (Figure 5A). This is expected, as the rules for sgRNA design are broader in this option. In line with this, the number of guides per motif was more diversified (up to 8), with the prevalence of 1–4 sgRNAs per motif. Average on- and off-target values as well as their distribution on histograms looked similar to the Cas9 mode. When the filters recommended for dCas9 mode were applied, i.e. motifs localized between −200 nt and + 100 nt relative to TSS were excluded, the number of found motifs went down to 873 and the number of designed guides decreased to 2815 (Figure 5B).

### Case 2: experimental validation of transCRISPR design

**Figure 6.**
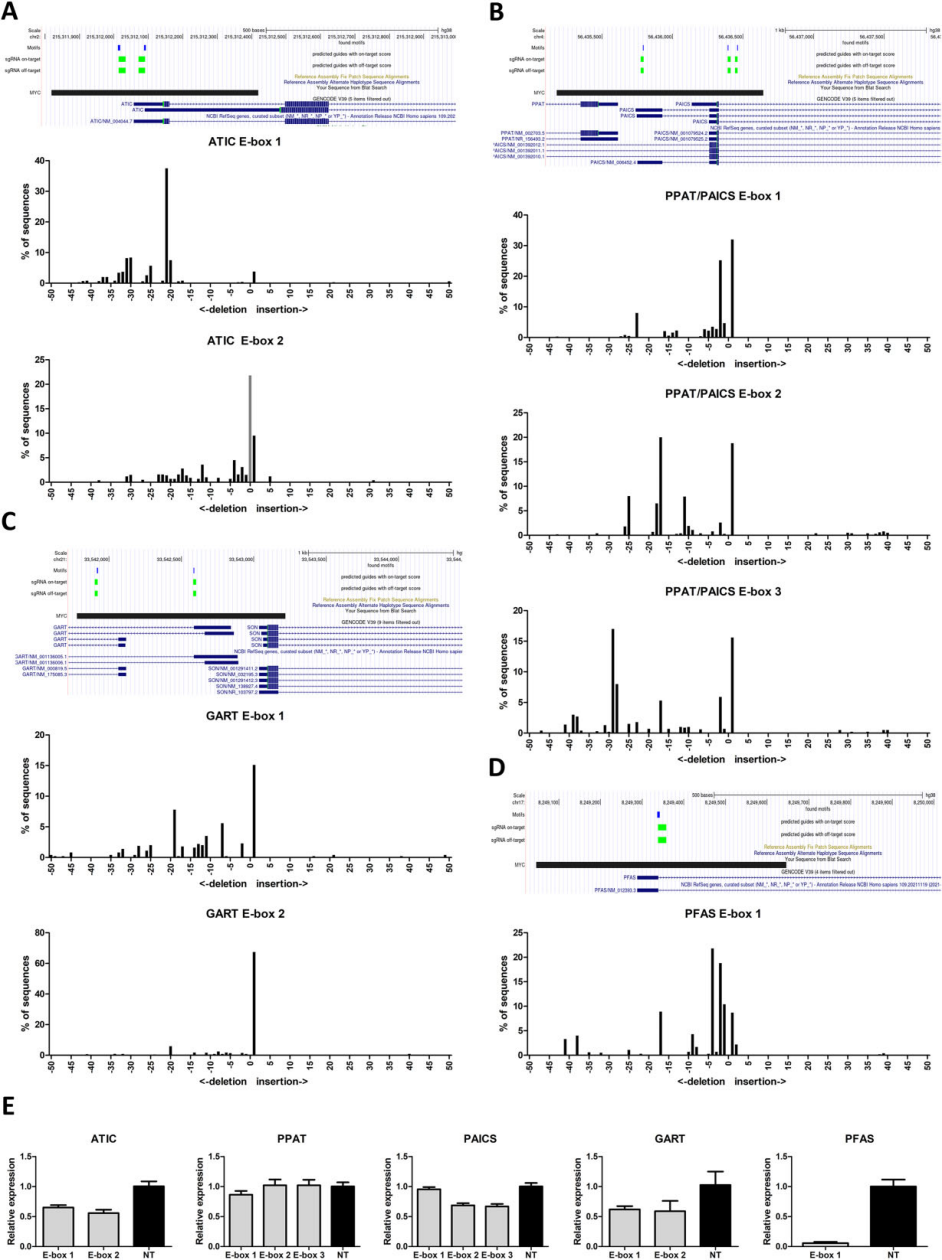
Experimental validation of sgRNAs designed by transCRISPR. (**A**–**D**) Genomic localization of identified E-box motifs (blue boxes) and targeting sgRNAs (green boxes) within the MYC-ChIP peaks (black boxes). Bar charts present results of TIDE analysis for sgRNAs targeting each E-box. ‘0’ means non-edited sequence (gray bar), ‘–‘ values represent deletion sizes, ‘+’ values represent insertion sizes. (**E**) Expression of genes localized near targeted E-boxes compared to control non-targeting (NT) sgRNAs (average of two NT constructs). Shown are the means and standard deviations from two independent experiments, each in triplicate.

To confirm that transCRISPR is able to design sgRNAs efficiently targeting sequence motifs, we took as an example genes involved in the purine biosynthesis pathway which are known to be regulated by MYC: *PPAT, GART, PFAS, PAICS* and *ATIC*. MYC-ChIP peaks proximal to these genes were retrieved from ENCODE data for K562 cells and used as the target sequence in transCRISPR, while the MYC motif matrix was obtained from Jaspar. For each MYC peak (*PPAT* and *PAICS* are localized in a head-to-head orientation with the common MYC peak within their promoter) transCRISPR identified 1–3 MYC binding motifs and designed sgRNAs targeting them (Figure 6A-D). sgRNAs showed good specificity (score 75–99) and moderate predicted efficiency (score 52–75). Designed sgRNAs were cloned into the lentiCRISPRv2_puro vector and used to transduce K562 cells (Supplementary Methods, Supplementary Table S1-3). Based on TIDE analysis (25), all sgRNAs resulted in efficient DNA editing (100% for all sgRNAs, except for ATIC E-box 2, which was 78%) and disruption of the E-box motifs (Figure 6A–D, Supplementary Figure S1). Importantly, CRISPR editing of E-box sequences decreased expression of the studied genes. The most pronounced effect was observed for PFAS. Disruption of the intergenic E-box localized between PPAT and PAICS did not affect either gene, while targeting the two intronic E-boxes within PAICS reduced expression of PAICS but not PPAT (Figure 6E).

This experiment demonstrated that transCRISPR enables design of efficient sgRNAs for targeted sequence motif disruption, which allows designing and performing experiments to answer biologically relevant questions.

## DISCUSSION

We developed transCRISPR to facilitate the study of specific sequence motifs. This is a unique tool that enables design of sgRNAs targeting a particular sequence in the region of interest and provides an important novel functionality for sgRNA design algorithms. The ability to disrupt or block sequence motifs can significantly facilitate research and understanding of their function. Importantly, transCRISPR offers wide range of functionalities and options to tailor the results to the user's needs and is applicable for small queries as well as design of genome-wide sgRNA libraries.

The biggest improvement introduced by transCRISPR is a versatile, efficient and well tested pipeline, which includes both custom code and available tools and algorithms. This pipeline is packed to be used in the queuing system, and the results are instantly displayed for the user in the form of comprehensive tables and diagrams.

For calculating off-targets we use Cas-OFFinder software, which is proven to work much more efficiently on GPUs. Unfortunately, we did not manage to obtain a dedicated webserver with powerful graphic cards, therefore we decided to increase the number of CPU cores available for calculations. If our server is overloaded with work, we will further increase the number of available cores.

We designed a consistent API for our webserver, but after multiple complex tests that took up to several days to complete, we decided to remove the possibility to submit query through API, to prevent overuse of our server. At the moment only check status and download results (in JSON format) options are available.

### Future plans

We plan to further expand the list of available reference genomes. We will also include more Cas9 variants, recognizing various PAM sequences. This will enable more comprehensive design of sgRNAs targeting specific motifs. We welcome all suggestions for improvement and development from the users via the contact details provided on the webpage.

## DATA AVAILABILITY

TransCRISPR is freely available at https://transcrispr.igcz.poznan.pl. Selected code snippets have been deposited on a github repository: https://github.com/tomaszwozniakihg/transcrispr_snippets, and on Zenodo, https://doi.org/10.5281/zenodo.7857006.

## Supplementary Material

gkad355_Supplemental_Files
