# Supplementary material for: TransCRISPR–sgRNA design tool for CRISPR/Cas9 experiments targeting specific sequence motifs
Source: Nucleic Acids Res. 2023 May 9;51(W1):W577–86. doi: 10.1093/nar/gkad355 (PMC10320084; doi:10.1093/nar/gkad355)
Supplement: gkad355_Supplemental_Files [file gkad355_supplemental_files.zip › Supplementary file NAR webserver.pdf]

SUPPLEMENTARY INFORMATION

TransCRISPR - sgRNA design tool for CRISPR/Cas9 experiments targeting DNA sequence motifs

Tomasz Woźniak, Weronika Sura, Marta Kazimierska, Marta Elżbieta Kasprzyk, Marta Podralska, Agnieszka Dzikiewicz-Krawczyk

SUPPLEMENTARY METHODS

Cell culture

K562 cells were cultured in RPMI medium (Lonza, Basel, Switzerland). HEK293T, used for virus production, were cultured in DMEM medium (Lonza). Media were supplemented with 10% fetal bovine serum (Sigma-Aldrich, Saint Louis, MO, US), 2mM L-glutamine and 1% penicillin/streptomycin (Biowest, Nuaillé, France). Cells were cultured in incubator under conditions: 5% CO_2, 37°C.

Transduction

sgRNAs targeting selected MYC binding motifs (E-boxes) were designed using transCRISPR and cloned into lentiCRISPR_v2 vector (Addgene #52961 (1)) along with two control non-targeting sgRNAs (Supplementary Table 1). Lentiviral particles were produced in HEK293T cells using 2nd generation packing plasmids and Calcium Phosphate Transfection Kit (Invitrogen, Carlsbad, CA, US). K562 cells were transduced as described previously (2). After selection with 3 µg/ml of puromycin for 96 h, cells were collected for DNA and RNA isolation 7 days post transduction.

Cutting efficiency analysis

DNA was isolated from transduced K562 cells using Gentra Puregene Cell Kit (Qiagen, Hilden, Germany). 500-800 nt DNA fragments containing regions targeted by selected sgRNAs were amplified by PCR (primers shown in Supplementary Table 2). Amplicons were subjected to Sanger sequencing (Genomed, Warsaw, Poland). Chromatograms from mutated and wild type samples were compared and analyzed with TIDE calculator (https://tide-calculator.nki.nl) (3) using indel size range of 50.

RT-qPCR

Total RNA was isolated from transduced K562 cells with Quick-RNA™ Miniprep Kit (Zymo Research, Irvine, CA, US). 500 ng of RNA was used for cDNA synthesis using QuantiTect® Reverse Transcription Kit (Qiagen). qPCR was performed with 5 ng of cDNA, PowerUp SYBR Green Master Mix (Applied Biosystems, Waltham, MA US) and primers presented in Supplementary Table 3. Expression of analyzed genes was normalized to *TBP* as a reference gene. All qPCR experiments were conducted in two independent biological replicates, each with three technical replicates.

Supplementary Table 1. Oligo sequences.

Oligo name	Sequence (sense)	Sequence (antisense)
**PFAS E-box1**	CACCGACGCGGTAACTATACTCACG	AAACCGTGAGTATAGTTACCGCGTC
**PPAT/PAICS E-box1**	CACCGAAAGCTGTATTTGCTGCACG	AAACCGTGCAGCAAATACAGCTTTC
**PPAT/PAICS E-box2**	CACCGAGAGTTTCGCGGCTCGCACG	AAACCGTGCGAGCCGCGAAACTCTC
**PPAT/PAICS E-box3**	CACCGCAGCCGCGCGGGCGCACACG	AAACCGTGTGCGCCCGCGCGGCTGC
**GART E-box1**	CACCGAGTAGCAAGTGAAAAGCACA	AAACTGTGCTTTTCACTTGCTACTC
**ATIC E-box1**	CACCGCTGTTTCCGGGCTTATCACG	AAACCGTGATAAGCCCGGAAACAGC
**ATIC E-box2**	CACCGAGCCCTCCTACCTGCGCACG	AAACCGTGCGCAGGTAGGAGGGCTC
**NT1**	CACCGACGGAGGCTAAGCGTCGCAA	AAACTTGCGACGCTTAGCCTCCGTC
**NT2**	CACCGATCGTTTCCGCTTAACGGCG	AAACCGCCGTTAAGCGGAAACGATC

Supplementary Table 2. Primer sequences for TIDE analysis.

TIDE primers	Primer F	Primer R
**PFAS E-box1**	CCCTGATCTTGCCAAGCAGA	CCCTCCCCAACTTTCAGGAC
**PPAT/PAICS E-box1**	ACATTCCTCTCGGATCCCCA	CGGAGACTGAAACGCGGATA
**PPAT/PAICS E-box2-3**	GCTCCAACTTCTGAGTCGCT	ACCTAACGGGCAAAGCAAGA
**GART E-box1**	GGCAAATAAAGCCGTATCAGAGG	TGGGTTTAGCATTTTGGCGC
**GART E-box2**	AGACACCCAAGCCTTAGCAC	CCAATTCGGTCTCTCGCCTT
**ATIC E-box1-2**	CTCACTTTGGGGTCGTGGG	AGGAGATTCTCGAATCGCAAGG

Supplementary Table 3. qPCR primer sequences.

Primer name	Primer F	Primer R
**TBP**	GCCCGAAACGCCGAATAT	CCGTGGTTCGTGGCTCTCT
**PFAS**	GTGAGTGGATCAAGCCCATC	GTAGACGGGACCTCCAACCT
**PPAT**	ACGCAGCTGGATGTACCG	CTAGTCACAATACCAGCACTCTCCT
**PAICS**	TCAAAAGAAATCCTGGTGTCAAG	CTGTGGGTCATTATTGGCATC
**GART**	CACAATGGAAGGCTTTCACC	AGACCACTGGCCTTCACAAC
**ATIC**	GCGTATCTCAGATGCCCTTG	AAATCCAGGGGCTCCATTTA

References

1. Sanjana,N.E., Shalem,O. and Zhang,F. (2014) Improved vectors and genome-wide libraries for CRISPR screening. *Nat Meth,* **11,** 783-784.

2. Kazimierska,M., Podralska,M., Zurawek,M., Wozniak,T., Kasprzyk,M.E., Sura,W., Losiewski,W., Ziolkowska-Suchanek,I., Kluiver,J.L., van den Berg,A., et al. (2022) CRISPR/Cas9 screen for genome-wide interrogation of essential MYC binding sites in cancer cells. *bioRxiv,* 2021.08.02.454734.

3. Brinkman,E.K., Chen,T., Amendola,M. and van Steensel,B. (2014) Easy quantitative assessment of genome editing by sequence trace decomposition. *Nucleic Acids Research,* **42,** e168-e168.

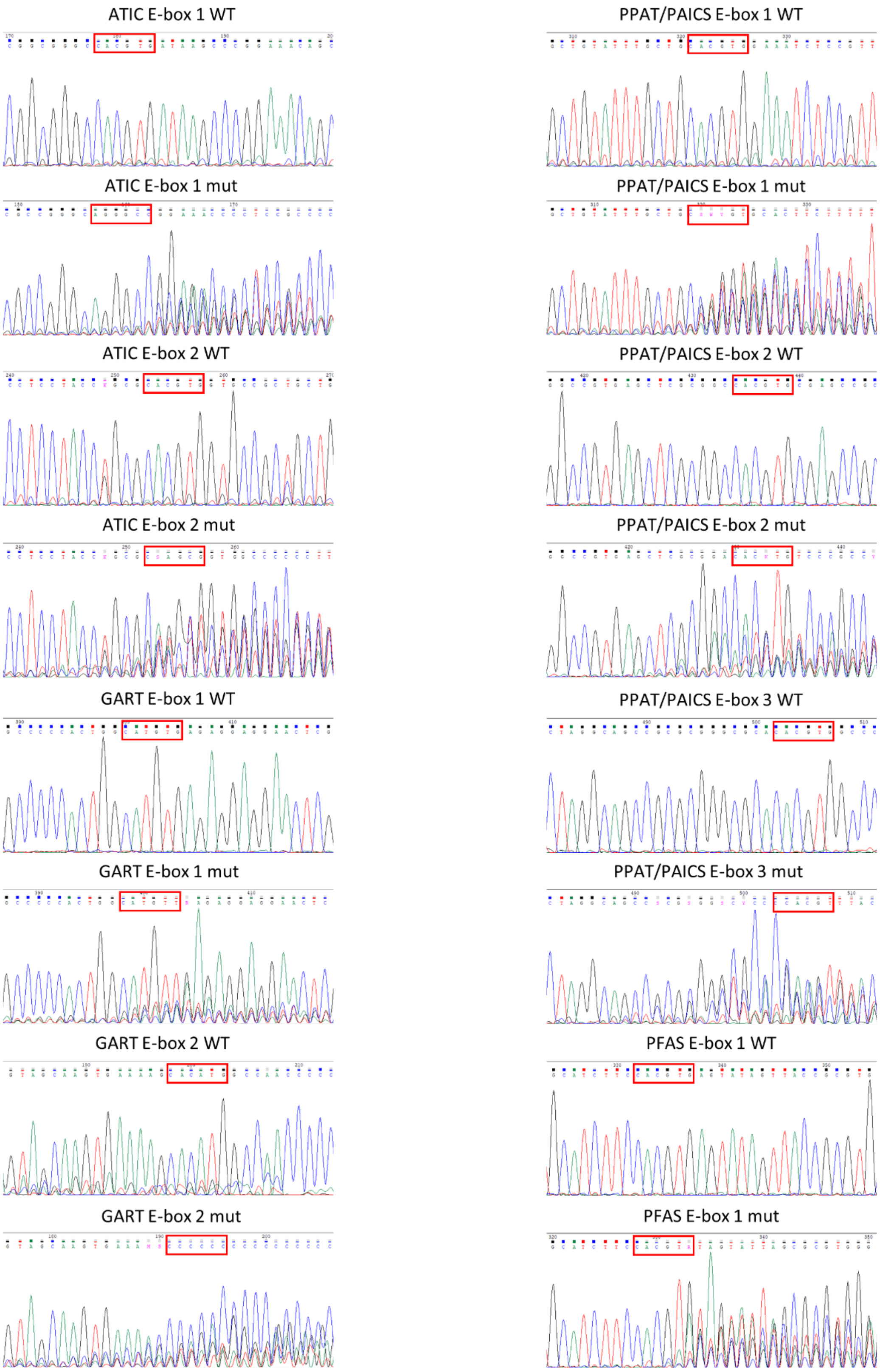

Supplementary Figure 1. Chromatograms from Sanger sequencing of wild type and CRISPR-edited samples. Red boxes indicate the targeted E-box motifs.
