# Supplementary material for: TransCRISPR–sgRNA design tool for CRISPR/Cas9 experiments targeting specific sequence motifs
Source: Nucleic Acids Res. 2023 May 9;51(W1):W577–86. doi: 10.1093/nar/gkad355 (PMC10320084; doi:10.1093/nar/gkad355)

MANUAL

1. What is transCRISPR?
2. Step 1 - Input: Genome selection.
3. Step 2 - Input: DNA sequence Motifs.
4. Step 3 - Input: Target region.
5. Step 4 - Input: Analysis parameters.
6. Output: Statistics.
7. Output: Found motifs, list of guides, on- and off-target scores.
   7.1 Motifs view
   7.2 Unique guides
   7.3. Downloading results
8. Output: Filter results.

NOTE for the user - In transCRISPR, the step you are currently in is marked in navy blue. You can check all the steps before loading your input.

1. What is transCRISPR?

transCRISPR is a website tool that enables design of CRISPR/Cas9 experiments for genome-wide targeting of user-defined sequences, for example transcription factors (TF) motifs. In this tool, the user provides information on DNA sequence motif and target region from the appropriate reference genome to design single guide RNA (sgRNA) oligonucleotides. transCRISPR is designed for both CRISPR/Cas9 and CRISPR/dCas9 systems. TransCRISPR comes with three preloaded examples, with various input formats for both motifs and target sequences, that can be used to get familiar with the tool.

2. Step 1 - Input: Genome selection.

In the first step of the analysis, the user has to select the appropriate reference genome. Currently, transCRISPR allows design for:

Human Dec. 2013 (GRCh38/hg38), Human Feb. 2009 (GRCh37/hg19)
Mouse Jun. 2020 (GRCm39/mm39)
Baboon Apr. 2017 (Panu_3.0/papAnu4)
Bonobo May 2020 (Mhudiblu_PPA_v0/panPan3)
C. elegans Feb. 2013 (WBcel235/ce11)
Cat Nov. 2017 (Felis_catus_9.0/felCat9)
Chicken Mar. 2018 (GRCg6a/galGal6)
Chimp Jan. 2018 (Clint_PTRv2/panTro6)
Cow Apr. 2018 (ARS-UCD1.2/bosTau9)
D. melanogaster Aug. 2014 (BDGP Release 6 + ISO1 MT/dm6)
Dog Oct. 2020 (Dog10K_Boxer_Tasha/canFam6)
Dog Mar. 2020 (UU_Cfam_GSD_1.0/canFam4)
Fugu Oct. 2011 (FUGU5/fr3)")
Gorilla Aug. 2019 (Kamilah_GGO_v0/gorGor6)
Horse Jan. 2018 (EquCab3.0/equCab3)
Lizard May 2010 (Broad AnoCar2.0/anoCar2)
Marmoset March 2009 (WUGSC 3.2/calJac3)
Orangutan Jan. 2018 (Susie_PABv2/ponAbe3)
Pig Feb. 2017 (Sscrofa11.1/susScr11)
Rat Nov. 2020 (mRatBN7.2/rn7)
Rat Jul. 2014 (RGSC 6.0/rn6)
Rhesus Feb. 2019 (Mmul_10/rheMac10)
S. cerevisiae Apr. 2011 (SacCer_Apr2011/sacCer3)
Sheep Nov. 2015 (Oar_v4.0/oviAri4)
Turkey Nov. 2014 (Turkey_5.0/melGal5)
X. tropicalis Nov. 2019 (UCB_Xtro_10.0/xenTro10)
X. tropicalis Jul. 2016 (Xenopus_tropicalis_v9.1/xenTro9)
Zebra finch Feb. 2013 (WashU taeGut324/taeGut2)
Zebrafish May 2017 (GRCz11/danRer11)

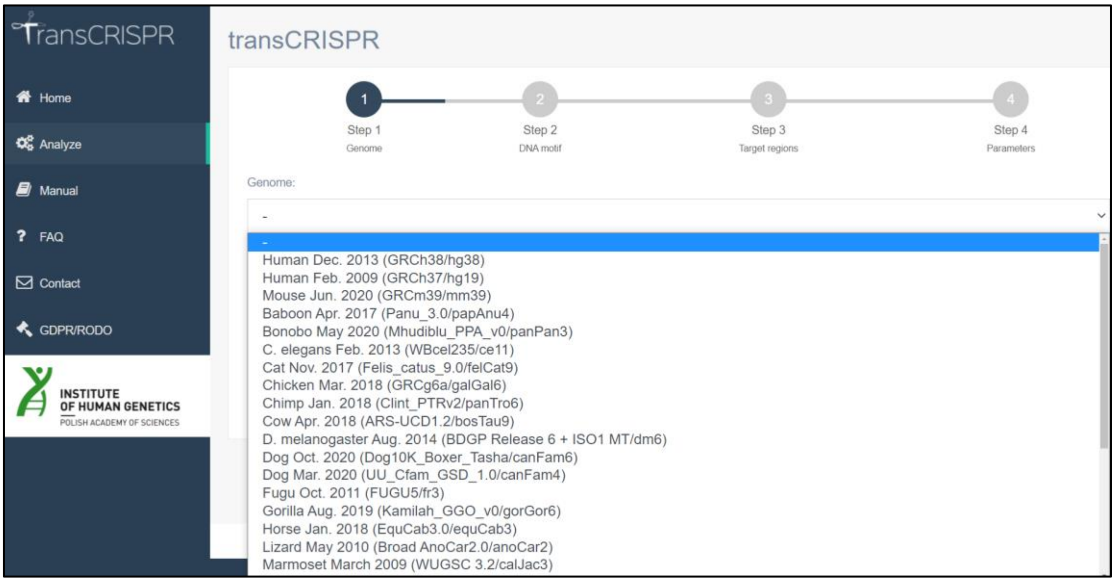

Currently, it is not possible to load a custom genome file.

3. Step 2 - Input: DNA Sequence Motifs

In Step 2, the user must provide the DNA motif of interest. transCRISPR allows for DNA motifs input in the form of a sequence [A] (FASTA format or coma separated, see examples below) or a matrix [B] (see available formats below). The user can choose whether to paste the input data into the window below [C] or to upload a pre-existing file [D, E]. Motifs must be provided as DNA nucleotides, degenerate nucleotides according to the IUPAC code are also allowed. Note that transCRISPR will also search for the provided motifs on the reverse strand of query target sequence. There is no length limitation (no minimum or maximum length) for DNA motif input. It is not required to have any previous knowledge if chosen DNA motifs are present in the target sequence/region (provided in Step 3).

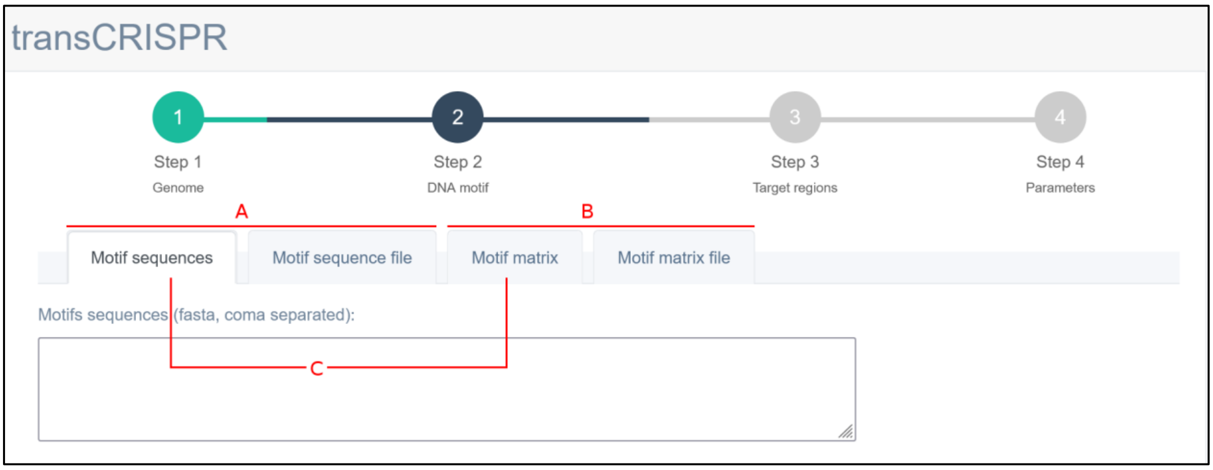

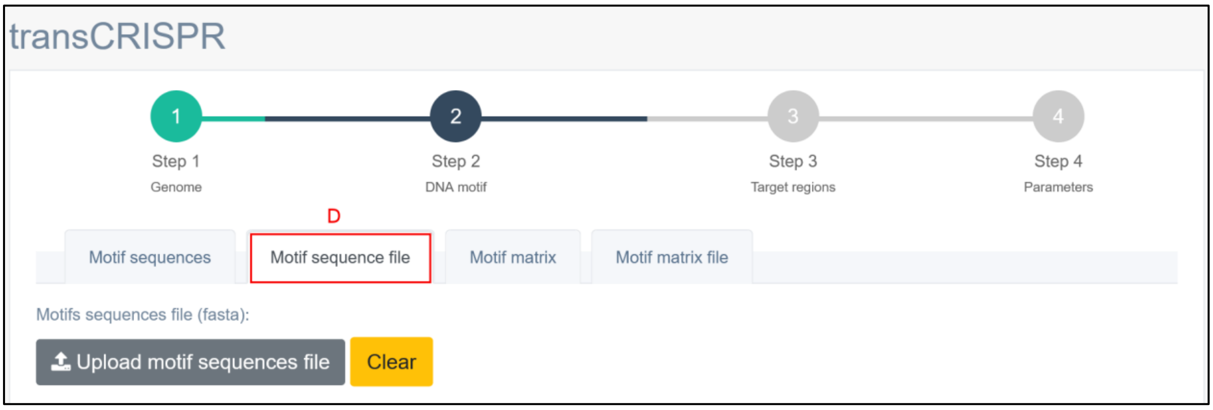

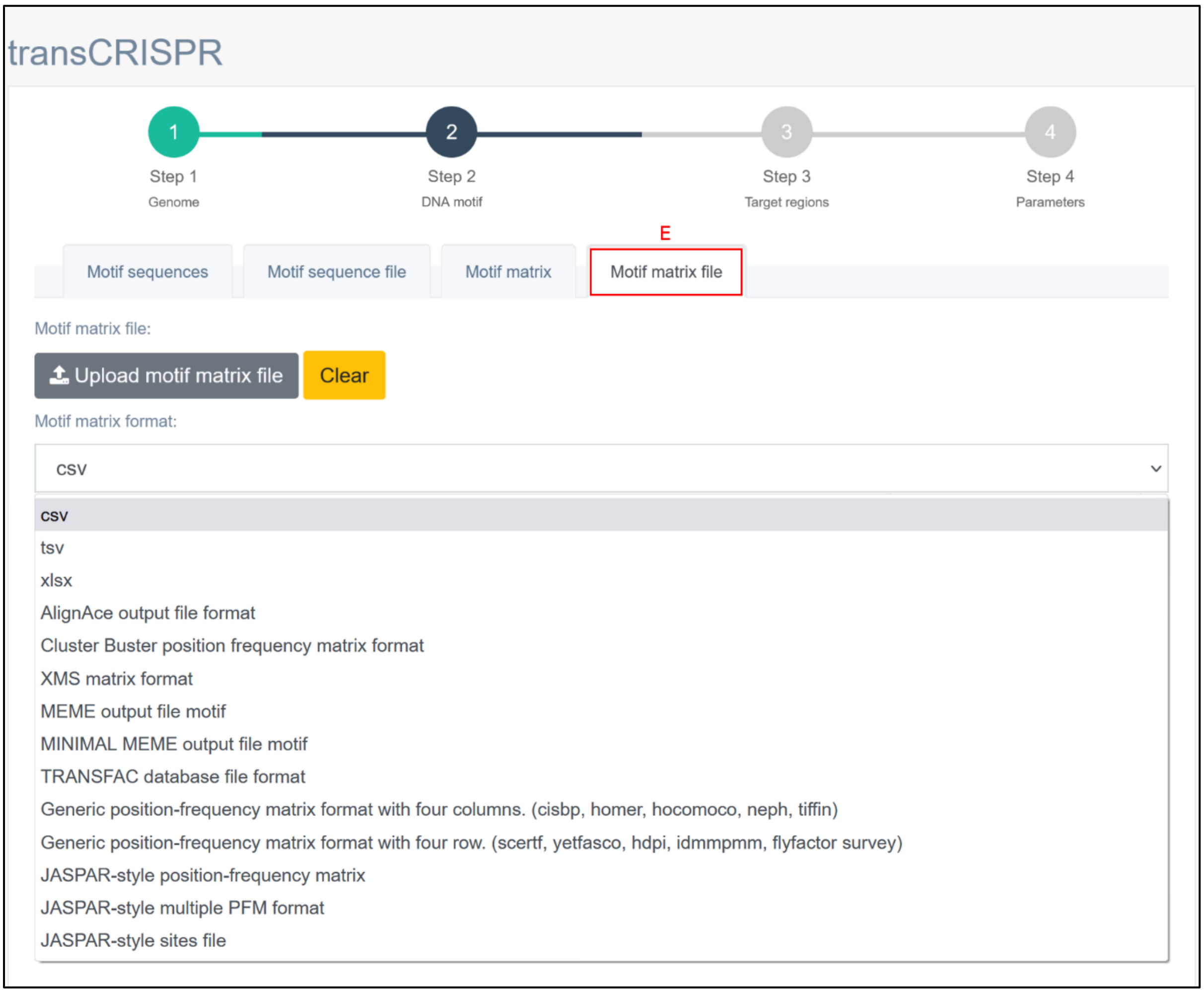

Example of FASTA format input:

>motif 1

BGGGGGRG

>motif 2

CGGGGGAG

>motif 3

GGGGGGGG

Example of coma-separated input:

BGGGGGRG, CGGGGGAG, GGGGGGGG, TGGGGGAG, TGGGGGGG

Available matrix motif formats:

csv, tsv, xlxs, AlignAce output file format, Cluster Buster position frequency matrix format, XMS matrix format, MEME output file motif, MINIMAL MEME output file motif, TRANSFAC database file format, Genomic position-frequency matrix format with four columns (crisbp, homer, hocomoco, neph, tiffin), Genomic position-frequency matrix format with four rows

(scertf, yetfasco, hdpi, idmmpmm, flyfactor survey), JASPAR-style position-frequency matrix, JAPSR-style multiple PFM format, JASPAR-style sites file

Click on the format name to be directed to the matrix motif example.

Example of csv motif matrix:

A,C,G,T
0.08,0.85,0.04,0.03
0.07,0.85,0.05,0.03
0.02,0.91,0.01,0.06
0.02,0.89,0.04,0.05
0.89,0.04,0.02,0.05
0.02,0.98,0.00,0.00
0.83,0.12,0.01,0.04

Example of tsv motif matrix:

A	C	G	T
0.08	0.85	0.04	0.03
0.07	0.85	0.05	0.03
0.02	0.91	0.01	0.06
0.02	0.89	0.04	0.05
0.89	0.04	0.02	0.05
0.02	0.98	0.00	0.00
0.83	0.12	0.01	0.04

Example of xlxs motif matrix:

	A	B	C	D
1	A	C	G	T
2	0.08	0.85	0.04	0.03
3	0.07	0.85	0.05	0.03
4	0.02	0.91	0.01	0.06
5	0.02	0.89	0.04	0.05
6	0.89	0.04	0.02	0.05
7	0.02	0.98	0.00	0.00
8	0.83	0.12	0.01	0.04

Transcription factor motifs can be found for example in JASPAR CORE database (https://jaspar.genereg.net/downloads/), where motifs can be downloaded in one of the provided matrix formats: JASPAR, MEME or TRANSFAC or Gene Transcription Regulation Database (GTRD, http://gtrd.biouml.org/). For targeting of miRNA binding sites or miRNA seed regions, databases such as miRbase (https://www.mirbase.org/), miRDB (https://mirdb.org/), STarMirDB (https://sfold.wadsworth.org/) or TargetScan (https://www.targetscan.org/) can be used to search for motifs of interest.

4. Step 3 - Input: Target region

In Step 3, the user must provide the target sequence in which transCRISPR will search for a given DNA motif. This may be a genomic sequence identified as a binding site for the TF e. g. by a chromatin immunoprecipitation experiment. transCRISPR allows for input sequence in different formats: [A] as genomic coordinates of a single sequence, [B] sequences as a text (FASTA and coma separated are acceptable), in [C] and [D] options, the user can upload a file with sequences or genomic coordinates, respectively. The file with genomic coordinates must contain: chromosome number, start and end and optionally the name of the sequence. Those can be separated by spaces, tabs or spaces and tabs.

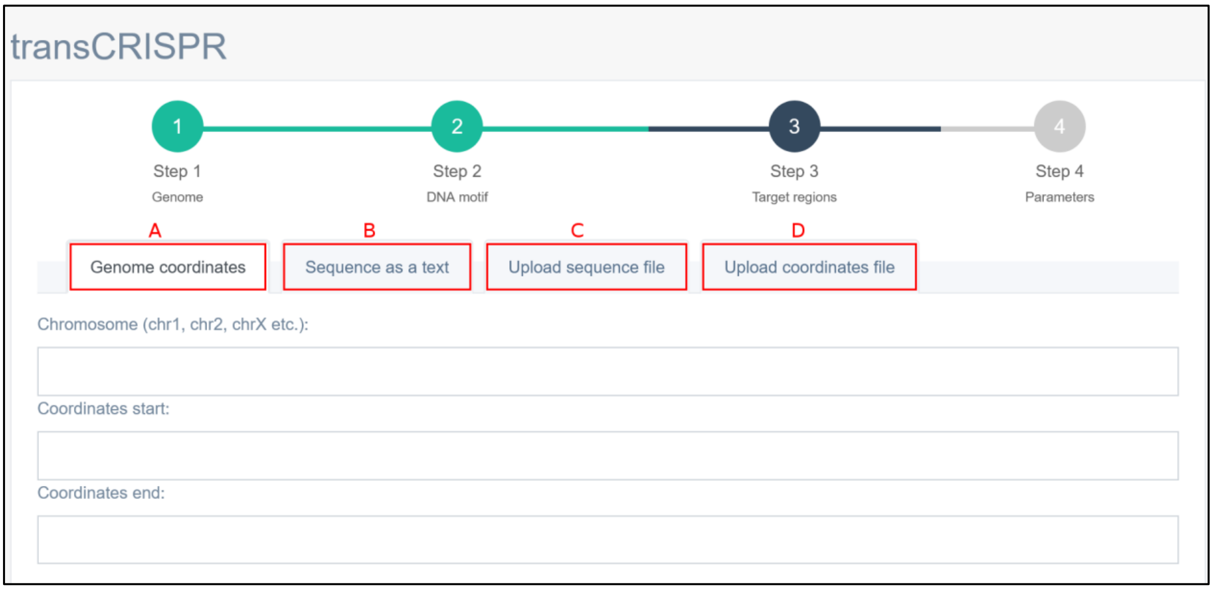

Example of file with genomic coordinates [D]:

chr2	264308	264501	sequence_name1
chr2	7017583	7018007	sequence_name2
chr8	580853	581274	sequence_name3
chr8	587997	588178	sequence_name4

If the user chooses to target motifs of transcription factors or other DNA-binding proteins, we recommend using Chip-Seq data for defining the target regions. Those can be found for example in Gene Expression Omnibus (GEO, https://www.ncbi.nlm.nih.gov/geo/), ENCODE (https://www.encodeproject.org/data-standards/chip-seq/) or Gene Transcription Regulation Database (GTRD, http://gtrd.biouml.org/). Visualization of Chip-Seq data for transcription factors binding sites can be found in UCSC Genome Browser (https://genome.ucsc.edu/) and also in GTRD.

Targeting all motifs in the genome

For targeting all motifs of interest in the genome, we recommend uploading genomic coordinates of the binding sites in the whole genome as the target sequence. Those can be obtained from the Chip-Seq data.

Targeting motifs for the specific gene region

For targeting motifs of interest within a specific gene region, for example gene promoter, we recommend uploading sequence or genomic coordinates spanning this region, e.g. in case of promoter: 1000 bp upstream of the transcription start site (TSS).

5. Step 4 - Input: Analysis parameters

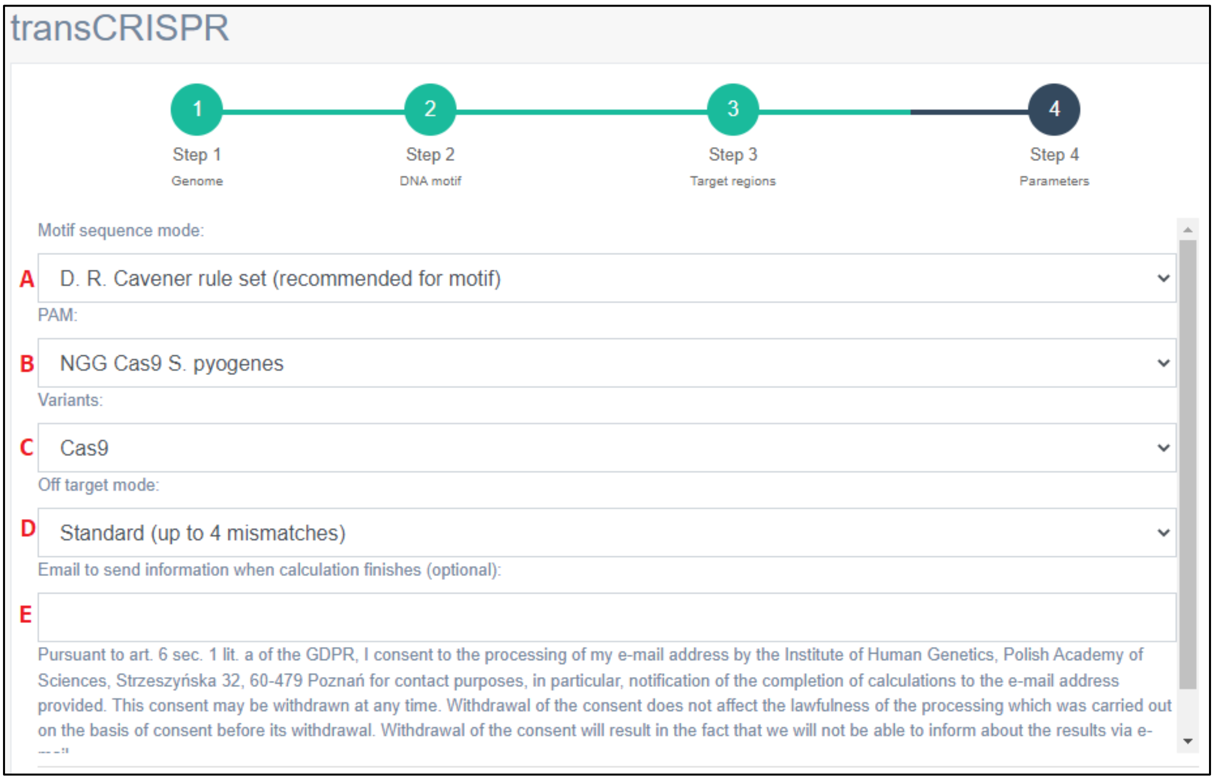

Motif sequence mode [A]:

Here, the user can choose the way in which DNA motifs will be generated for analysis. If in Step 2 the user provides motif as a sequence or sequence file, transCRISPR will analyze all motif sequences separately (i.e. it will search for all given sequences). If the user provides motif as a motif matrix or matrix file, different modes of constructing motifs based on the matrix will be available (see below): 1) the D. R. Cavener rule set, 2) frequency for nucleotide - here transCRISPR will take into the motif every nucleotide which covers at least x% at a given position; or 3) select the most frequent nucleotides – here transCRISPR will include in the motif the most common nucleotides at a given position, which together constitute at least x%.

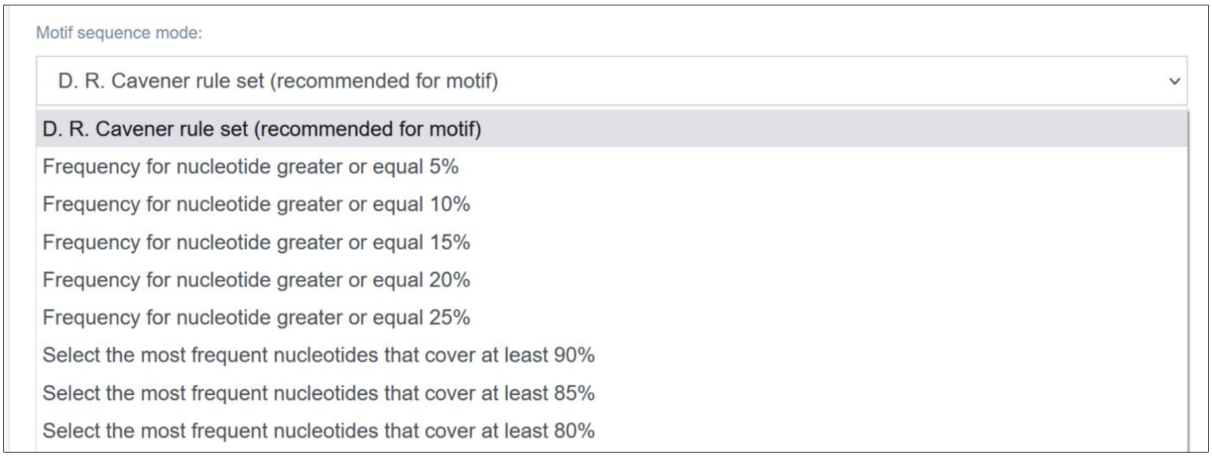

PAM [B]:

Here, the user may choose from three variants of PAM (protospacer adjacent motif) sequences recognized by SpCas9: NGG, NGA and NGCG. TransCRISPR searches for PAM on both plus and minus strand of the query target sequence.

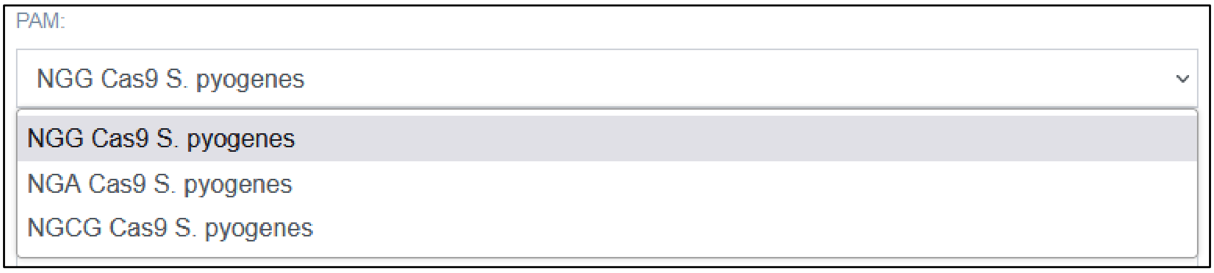

Variants [C]:

In Step 4, the user can choose to design sgRNA oligonucleotides either for CRISPR/Cas9 or CRISPR/dCas9 (catalytically-inactive, dead-Cas9) of *Streptococcus pyogenes* Cas9 protein (SpCas9). Note that the design rules for Cas9 and dCas9 are different. See the manuscript and FAQ for more details. The third option, ‘Custom’, allows to define the maximum distance of PAM (in bp) from the motif. For example if the maximal distance will be set as 30 this means that PAM will be searched and guides designed up to 30 bp upstream and 30 bp downstream from the motif. At least 1 nt of PAM need to be included in the custom search range to be found by transCRISPR in the custom mode.

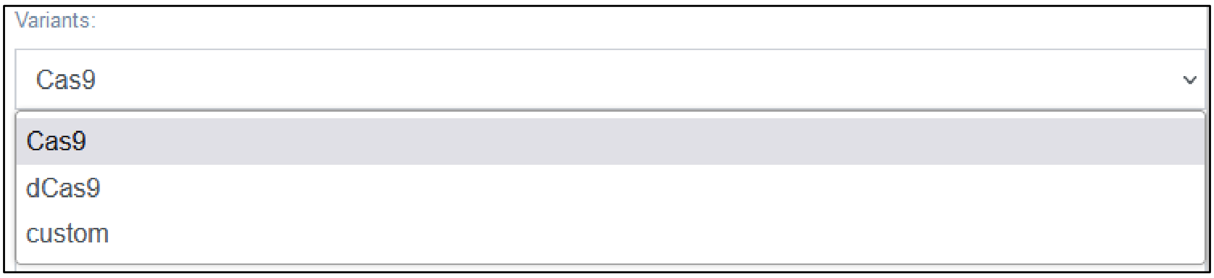

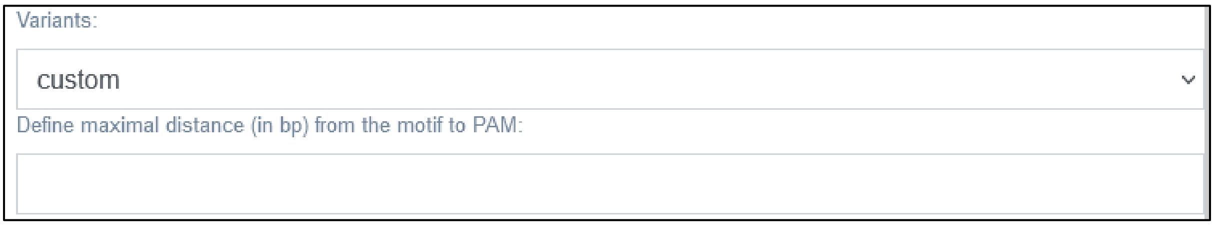

Off-target mode [D]:

The user may choose to analyze off-targets in two modes: standard (up to 4 mismatches) or rapid (up to 3 mismatches).

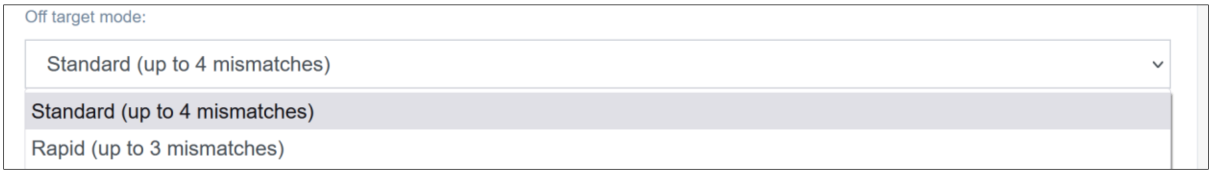

E-mail [E]:

The user may choose to be informed via e-mail when the analysis is finished. This is not required for the analysis to start, but may be useful, especially for bigger tasks. E-mail will be sent to the user from the following address: tpsic@man.poznan.pl and will contain the link to the results page. If an e-mail will be provided, the user must consent to the processing of the e-mail address (see below), otherwise the e-mail cannot be sent.

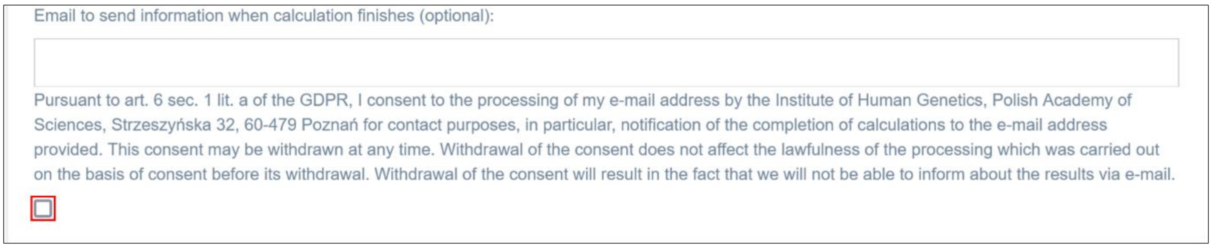

View our General Data Protection Regulation (GDPR) here.
If the user does not wish to provide their e-mail, the link generated after starting the analysis can be used to access the results for 7 days.

6. Output: Statistics

The upper panel of transCRISPR results [A] contains the total number of motifs and guides found in a given query, average number of guides found per motif and average off-target and on-target scores. Query details are summarized in [B].

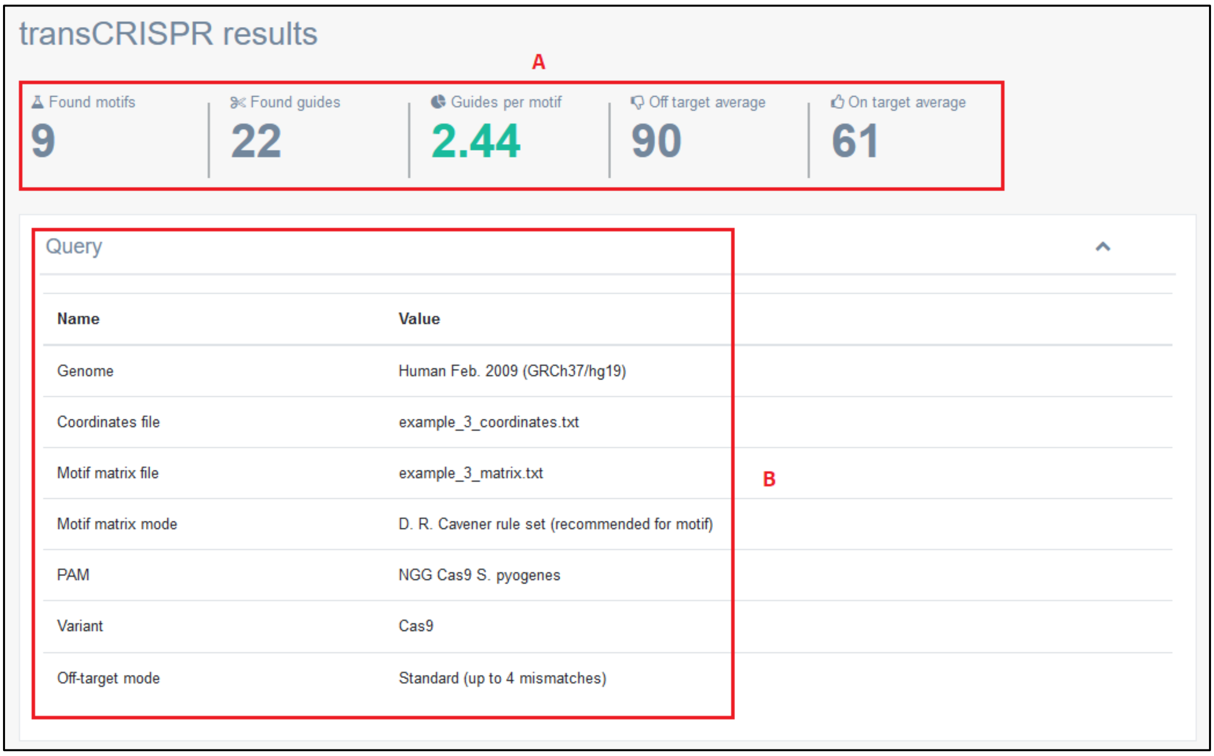

The next panel in transCRISPR results provides the charts: on-target and off-target graphs, distribution of guides per motif and genomic localization distribution of motifs. Using ≡ icon the user can open options available for each chart. Charts can be downloaded as png, jpeg, pdf or svg file. Data corresponding to each chart can be downloaded as csv, xls or the data table can be viewed on site. Charts will be updated if filters are set.

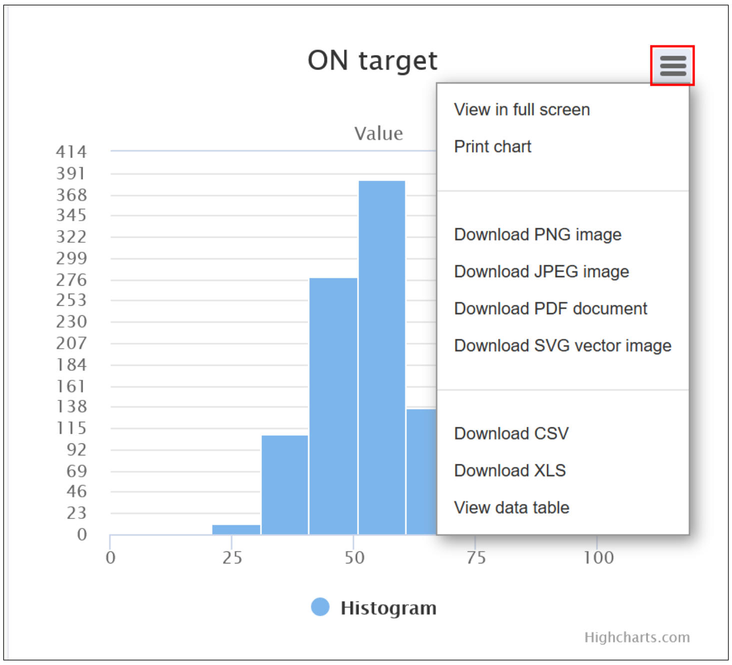

On-target and off-target scores are calculated based on the Rule Set 2 and CFD scores, respectively, both described in [PMID 26780180]. The scores are visualized on histograms for a general overview, while detailed scores for each guide are present in the results table - for more details, go to 7. Output: List of guides, on- and off-target scores.

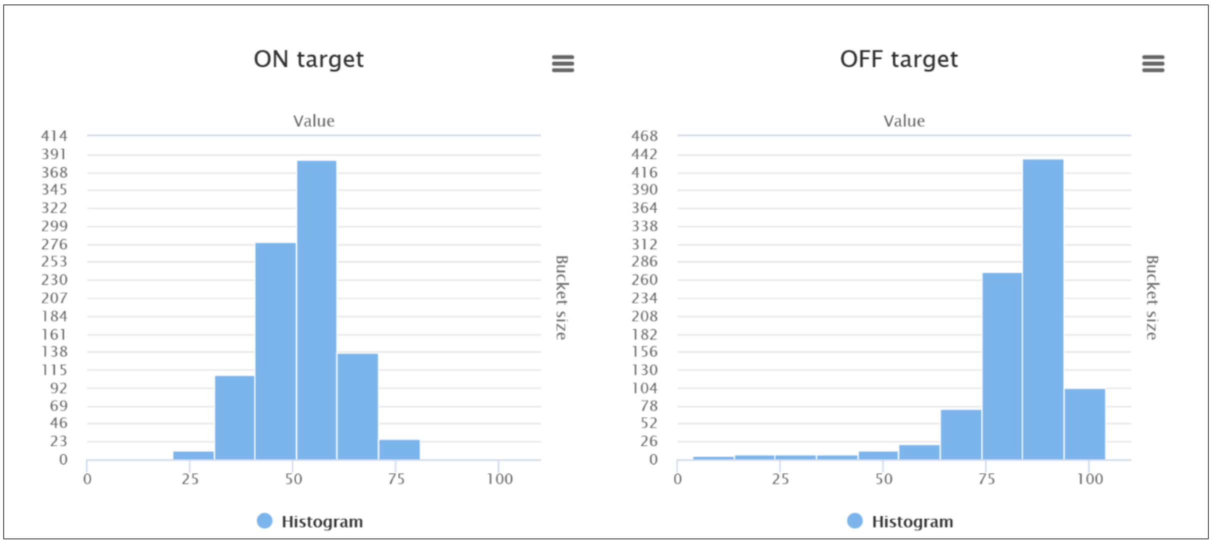

Distribution of guides per motif shows the statistics on the number of guides designed for the identified motifs. The chart below should be read as follows: no guides were designed for 40.7% of motifs (blue), one guide was designed for 33.7% of motifs (black), two guides were designed for 16.4% of motifs (green) etc.

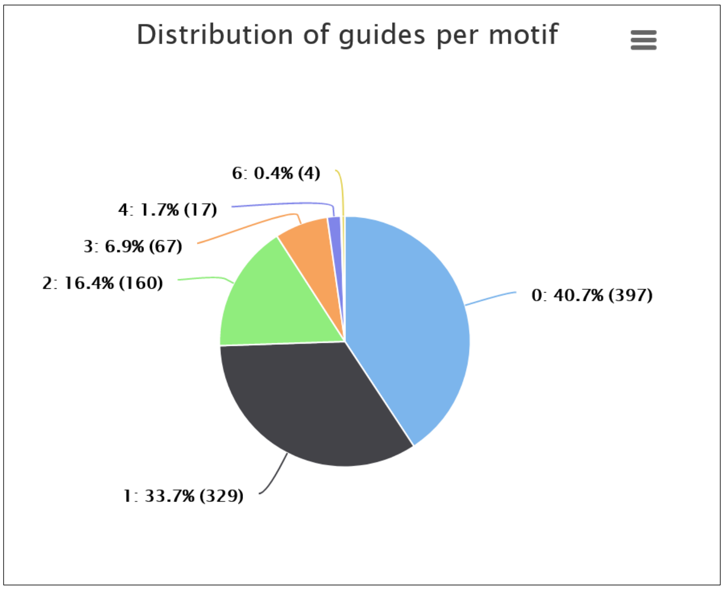

Genomic localization distribution of motifs shows where in the genome the identified motifs are localized: coding exons, non-coding exons, introns or intergenic. Note that if the user provides the target sequence in Step 3 - the graph will not show the genomic distribution. This option is available only if the genomic coordinates are provided.

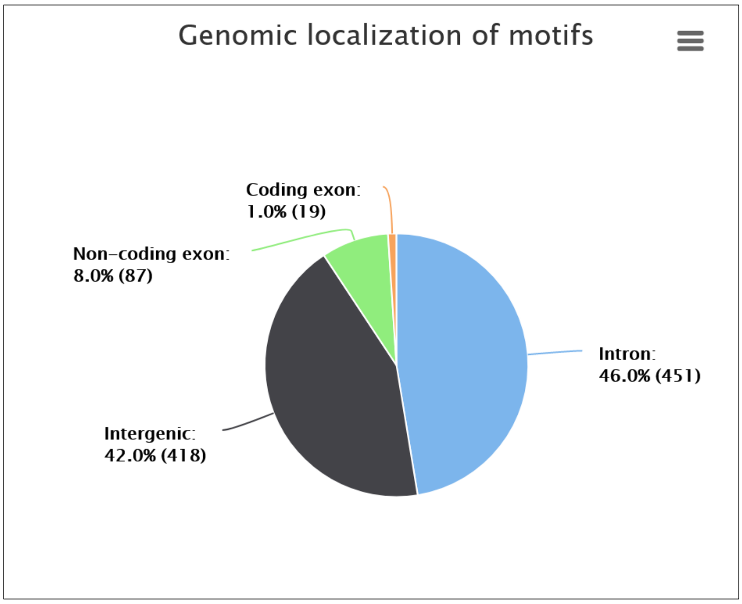

7. Output: List of guides, on- and off-target scores

Next part of the results is the “Found motifs and guides” section. Here transCRISPR shows in detail DNA motifs that were found and if any sgRNAs were designed for those motifs. TransCRISPR presents results as a table with the list of identified DNA motifs Motifs or as a table with unique guides Unique guides .

7.1 Motifs view

If the user chooses Motifs , the table with found motifs is presented and for each particular motif a list of guides is shown.

Sequence ID

Indicates the sequence in which the motifs/guides are found (in case more than one sequence/genomic coordinates were provided). By default they are named by chromosome and consecutive seq numbers. If the user provided sequences in FASTA format with sequence names, original names of sequence are retained.

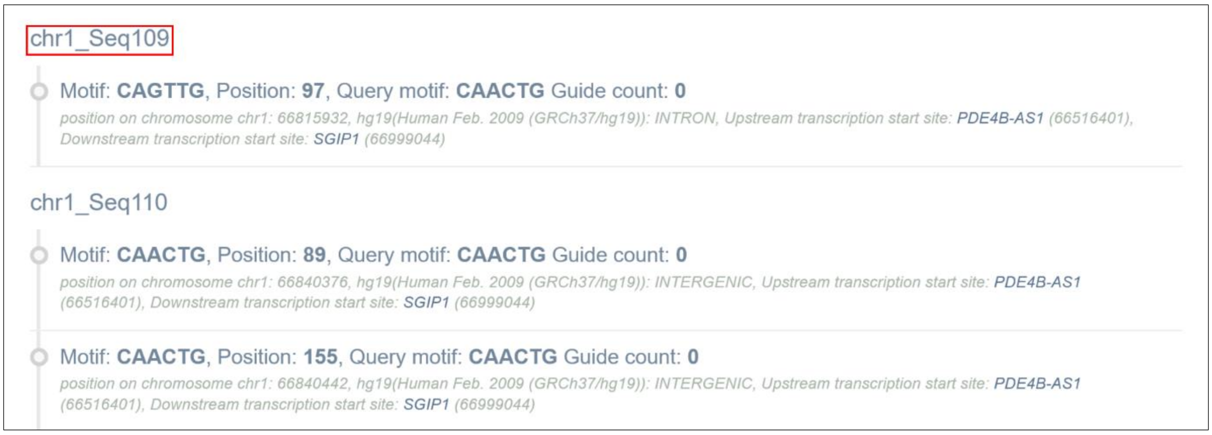

Motif information

Next, the user can find information on motif sequence (note that motif sequence can be shown in reverse complement if motif was found on the minus strand), motif position in the query sequence, query motif sequence and number of guides designed for this motif. Information in italics provides position on chromosome with the start coordinate, genome, genomic localization of the motif (coding or non-coding exons, introns or intergenic) and transcription start site (TSS) of the closest up- and downstream gene. In case of genes with multiple

transcripts, TSS position for the longest transcript is provided. Note that information in italics are available only if the user provides genomic coordinates of the target sequence.

Motif: **CAGTTG**, Position: **49**, Query motif: **CAACTG** Guide count: **1**

position on chromosome chr1: 67896595, hg19(Human Feb. 2009 (GRCh37/hg19)): INTERGENIC, Upstream transcription start site: SERBP1 (67896085), Downstream transcription start site: GADD45A (68150884)

Guides information

Order of guides for particular motifs depends on the filter setting.

Strand

In the first column, the user receives information about the strand on which the PAM sequence and sgRNA were found: plus or minus (+/-). Plus strand corresponds to the reference genome sequence if coordinates were used as an input, or to the sequence provided by the user as a text.

Strand	Relative position	Sequence	Off target 0-1-2-3-4	Off target score	On target score
+	56	AGTCCGAGCTGCTCAGTTGC	0-0-0-7-143 Details	87	37

Relative Position

Nucleotide number indicates the beginning of guide 5' - 3' counting from starting position of the query sequence.

Strand	Relative position	Sequence	Off target 0-1-2-3-4	Off target score	On target score
+	56	AGTCCGAGCTGCTCAGTTGC	0-0-0-7-143 Details	87	37

Sequence

In the third column of the table, transCRISPR shows the sgRNA sequences.

Strand	Relative position	Sequence	Off target 0-1-2-3-4	Off target score	On target score
+	56	AGTCCGAGCTGCTCAGTTGC	0-0-0-7-143 Details	87	37

Off-targets

In the fourth column, the user receives information on off-targets. “Off targets 0-1-2-3-4” indicates off-targets with the certain number of mismatches. For example, 0-0-6-8-169 means 0 off-targets with 0 mismatches (other than itself), 0 off-target with 1 mismatch, 6 off-targets with 2 mismatches, 8 off-targets with 3 mismatches, 169 off-targets with 4 mismatches.

Strand	Relative position	Sequence	Off target 0-1-2-3-4	Off target score	On target score
+	56	AGTCCGAGCTGCTCAGTTGC	0-0-0-7-143 Details	87	37

Detailed list of off-targets can be accessed and downloaded by clicking on the “Details” button. The CFD score for a single off-target is 0-1. 1 means 100% match. Details for off-targets up to 3 mismatches are displayed.

Off targets — Download off targets

Chromosome: **chr21**	Strand: **+**	Position: **46419744**	Sequence: **AGTCCGtGCTGCgCAGgTGCTGG**	Mismatches: **3**	CFD score: **0.013**
Chromosome: **chr2**	Strand: **-**	Position: **53098041**	Sequence: **AGTCCcAGCTaCTCAGaTGCTGG**	Mismatches: **3**	CFD score: **0.364**
Chromosome: **chr2**	Strand: **-**	Position: **24857700**	Sequence: **AGTCCcAGCTaCTCAGTTGgGGG**	Mismatches: **3**	CFD score: **0.040**
Chromosome: **chr1**	Strand: **-**	Position: **7725096**	Sequence: **ccTCCGAGCTGCTCAGcTGCAGG**	Mismatches: **3**	CFD score: **0.419**
Chromosome: **chr22**	Strand: **+**	Position: **47460154**	Sequence: **AGTgCaAGCTGCTCAGTTcCCGG**	Mismatches: **3**	CFD score: **0.224**
Chromosome: **chr11**	Strand: **-**	Position: **11400062**	Sequence: **gGTCaGAGCTGCTCAGaTGCTGG**	Mismatches: **3**	CFD score: **0.305**
Chromosome: **chr11**	Strand: **+**	Position: **62752297**	Sequence: **AGTCCGAGCTGCTCtGTgGgGGG**	Mismatches: **3**	CFD score: **0.004**

It is possible to save off-targets details for a particular guide as an excel sheet. Localization, sequence, number of mismatches and CFD score will be provided for each off-target.

	A	B	C	D	E	F
1	Chromosome	Strand	Position	Sequence	Mismatches	CFD score
2	chr21	+	46419744	AGTCCGtGCTGCgCAGgTGCTGG	3	0,013
3	chr2	-	53098041	AGTCCcAGCTaCTCAGaTGCTGG	3	0,364
4	chr2	-	24857700	AGTCCcAGCTaCTCAGTTGgGGG	3	0,04
5	chr1	-	7725096	ccTCCGAGCTGCTCAGcTGCAGG	3	0,419
6	chr22	+	47460154	AGTgCaAGCTGCTCAGTTcCCGG	3	0,224
7	chr11	-	11400062	gGTCaGAGCTGCTCAGaTGCTGG	3	0,305
8	chr11	+	62752297	AGTCCGAGCTGCTCtGTgGgGGG	3	0,004

Off/On-target score

The off-target CFD score indicates the predicted off-target activity of the sgRNA. Off-target score range is 0-100, the higher, the better. 100 means that no off-targets with up to 4 mismatches were found. The on-target score indicates the probability of successful cleavage for a particular guide. On target score range is 0-100, the higher, the better. Off- and on-target scores are colored: 0 : ≤ 30 – red, >30 : ≤50 – yellow, >50 : ≤100 – green.

Strand	Relative position	Sequence	Off target 0-1-2-3-4	Off target score	On target score
+	56	AGTCCGAGCTGCTCAGTTGC	0-0-0-7-143 Details	87	37

7.2. Unique guides view

If the User presses Unique guides, the table will present unique guides. This option is important in case two motifs partially overlap in the sequence and as a result, some sgRNAs will target them both. This option helps to avoid generating sgRNA libraries with duplicate guides. As previously, the user receives information about strand, position, and sequence of guides. Off/On-target scores are also shown.

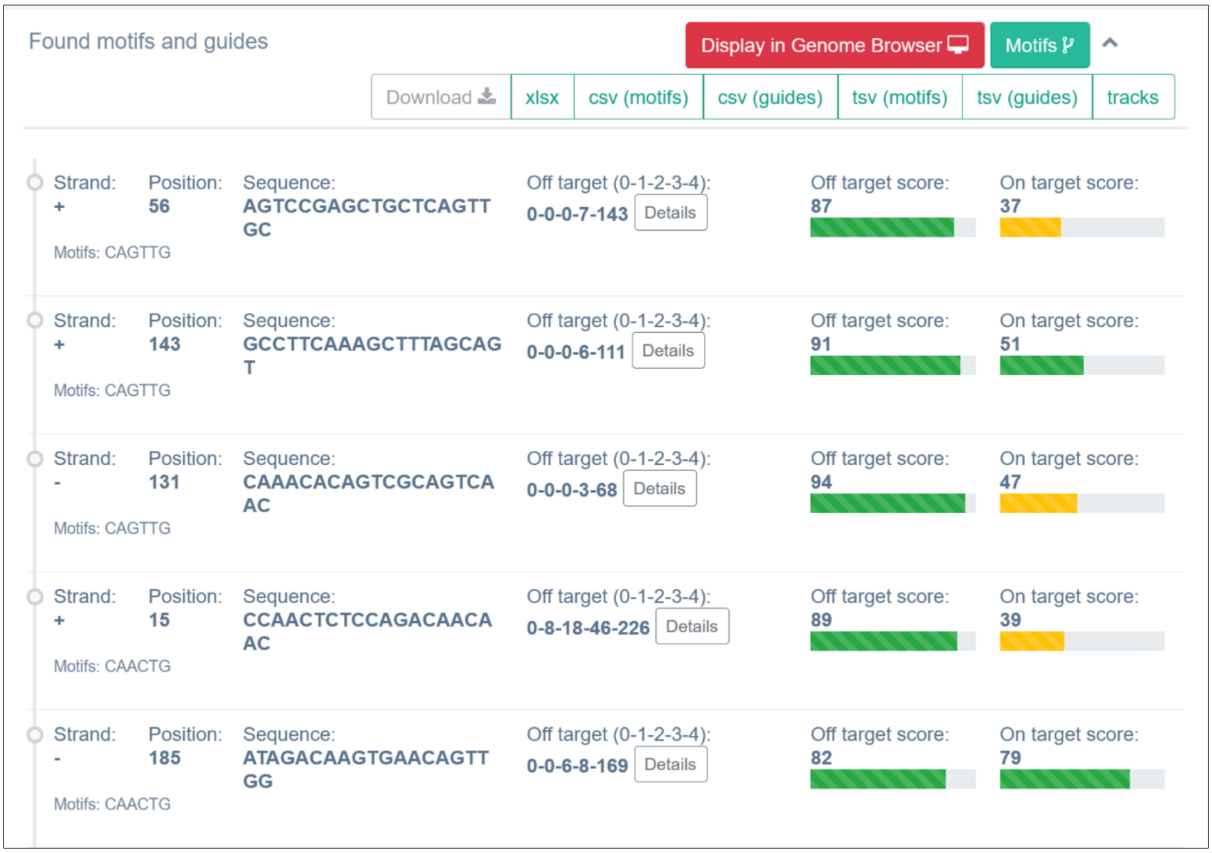

7.3. Downloading results

TransCRISPR results can be downloaded in one of the provided formats: xlsx, csv, tsv [A] or as tracks [B]. Using the button “Display in Genome Browser” [C], the user will be redirected to UCSC Genome Browser, where guides and motifs will be displayed as tracks. Note that if the user provides the query target as sequence, “Display in Genome Browser” and tracks download will not be available. Genome Browser options are available only for human and mouse genomes.

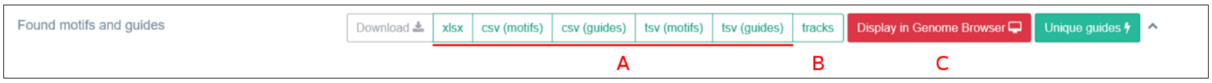

xlsx format

In the first Excel sheet the user receives information on input data used for analysis.

	A	B
1	**name**	**value**
2	Genome	Human Dec. 2013 (GRCh38/hg38)
3	Search sequence	ATATATATATATATATATATGCGATCATCATCATCATCATCAAAATGCGAAAATAT ATTTATATATATATATATGCGATCATCCATCATC
4	Motif sequences	AAAATGCGAAA
5	Motif matrix mode	Analyze all sequences separately
6	PAM	NGCG Cas9 S. pyogenes
7	Variant	Custom (Search length: 14)
8	Off target mode	Rapid (up to 3 mismatches)

The second Excel sheet provides information for each motif found: coordinates of target sequence and motif, no. of sgRNAs found per motif, name and TSS position of the closest gene up- and downstream, and genomic localization of the motif. Motifs are named in the format: target sequence name_motif sequence_no. of consecutive motifs with this sequence in the target region.

	A	B	C	D	E	F	G	H	I	J	K	L
1	**DNA motif**	**chr**	**Seq start**	**Seq end**	**motif start**	**motif end**	**no. of sgRNAs**	**gene upstream**	**gene upstream TSS**	**gene downstream**	**gene downstream TSS**	**genomic localization**
2	sequence1_CACGTG_1	chr2	264308	264501	264395	264401	3	FAM110C	46505	SH3YL1	264824	Intron
3	sequence2_CATGTG_2	chr2	7017583	7018007	7017641	7017647	1	CMPK2	7006766	RSAD2	7017908	Intergenic
4	sequence2_CACATG_1	chr2	7017583	7018007	7017772	7017778	1	CMPK2	7006766	RSAD2	7017908	Intergenic
5	sequence2_CATGTG_1	chr2	7017583	7018007	7017774	7017780	1	CMPK2	7006766	RSAD2	7017908	Intergenic
6	sequence3_CACATG_1	chr8	580853	581274	580965	580971	4	TDRP	495780	ERICH1	681224	Intron
7	sequence3_CACGTG_1	chr8	580853	581274	581004	581010	2	TDRP	495780	ERICH1	681224	Intron
8	sequence3_CATGTG_1	chr8	580853	581274	581089	581095	2	TDRP	495780	ERICH1	681224	Intron
9	sequence3_CACATG_2	chr8	580853	581274	581128	581134	3	TDRP	495780	ERICH1	681224	Intron
10	sequence4_CACGTG_1	chr8	587997	588178	588171	588177	5	TDRP	495780	ERICH1	681224	Intron

The third Excel sheet provides information about unique sgRNAs designed for the identified motifs: targeted motif, sgRNA sequence and strand, localization, off- and on-target score. sgRNA name is constructed as follows: motif name_sg1, 2, 3… - sgRNAs targeting the same motif are numbered consecutively.

	A	B	C	D	E	F	G	H	I
1	**DNA motif**	**sgRNA name**	**sgRNA sequence**	**strand**	**chr**	**sgRNA start**	**sgRNA end**	**CFD off-target score**	**on-target score**
2	sequence1_CACGTG_1	sequence1_CACGTG_1_sg3	CAGCGCGAGTCTCGCGCACG	-	chr2	264397	264417	98	57
3	sequence1_CACGTG_1	sequence1_CACGTG_1_sg2	CGCGAGTCTCGCGCACGTGG	-	chr2	264394	264414	99	52
4	sequence1_CACGTG_1	sequence1_CACGTG_1_sg1	GCGAGTCTCGCGCACGTGGC	-	chr2	264393	264413	98	45
5	sequence2_CATGTG_2	sequence2_CATGTG_2_sg1	GCTTGATGTAGTTACACATG	-	chr2	7017641	7017661	92	64
6	sequence2_CACATG_1	sequence2_CACATG_1_sg1	TCAACTTTCAGTTTCACATG	+	chr2	7017758	7017778	80	60
7	sequence3_CACATG_1	sequence3_CACATG_1_sg1	GAGTCAGCTCTGTCCATGTG	-	chr8	580965	580985	84	68
8	sequence3_CACATG_1	sequence3_CACATG_1_sg4	CCTCCTGTGAGCACCCCACA	+	chr8	580949	580969	84	59
9	sequence3_CACATG_1	sequence3_CACATG_1_sg3	GTGAGTCAGCTCTGTCCATG	-	chr8	580967	580987	78	53
10	sequence3_CACATG_1	sequence3_CACATG_1_sg2	TGAGTCAGCTCTGTCCATGT	-	chr8	580966	580986	85	49

The same information is provided in csv, tsv formats.

Tracks can be downloaded in the .bed format for future use in the UCSC Genome Browser. Optionally, the results can be directly displayed in Genome Browser by clicking on Display in Genome Browser

Genome Browser options are available only for human and mouse genomes.

Example of the view in UCSC Genome Browser:

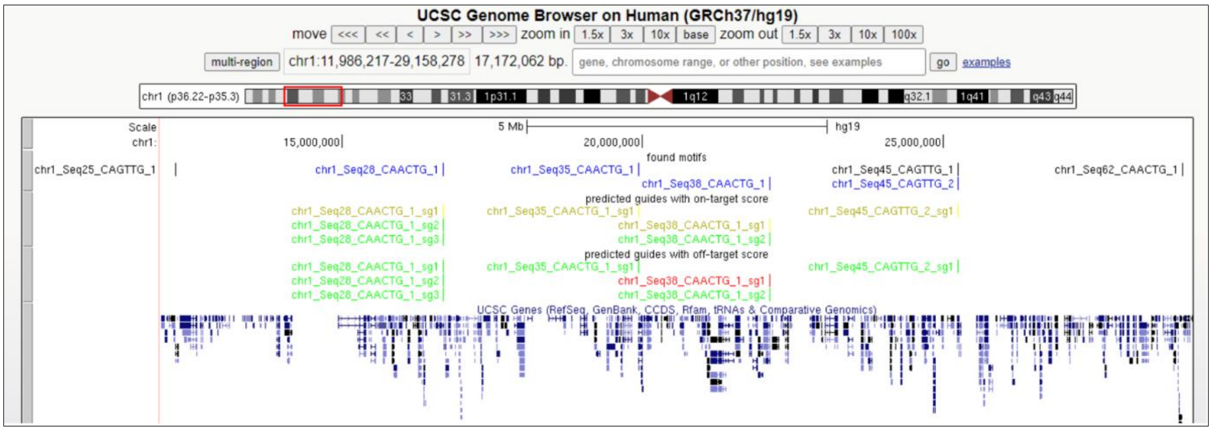

Legend:
Motifs in blue - DNA motifs for which guides were designed
Motifs in black - DNA motifs for which guides were not designed
sgRNA on-target – designed guides, colored by on-target score
sgRNA off-target – designed guides, colored by off-target score

For sgRNA color details go to section Off/On-target score.

8. Output: Filter results

The user may filter the results obtained in the transCRISPR, using several filters.

Remove filters button clears all filters.

When filtering by motifs [A] the user may decide to display the motifs by their localization in the provided target input, that is in coding exon / intron / intergenic / non-coding exon and localization plus or minus to TSS (in base pairs, bp). If the user decided to design guides for Cas9 system it is recommended to choose those guides that were designed for motifs located in non-coding exons, introns and intergenic. This is because targeting motifs in coding exons will likely lead to disruption of the encoded protein and the obtained results will not be specific for disruption of DNA motif of interest. If the user decided to design guides for dCas9 system it is recommended to exclude motifs located between minus 200 nt to plus 100 nt relative to TSS. That is because this window is known to be the most effective for CRISPRi and it may be hard to distinguish if the observed effect is caused by targeting of DNA motif of interest or due to transcription inhibition caused by dCas9 in the given region. Filters related to motifs are available only if the user provided genomic coordinates of the target region as an input.

When filtering by guides [B] the user can choose the off- and on-target cut-off, display only the chosen number (x) of the best guides per motif based on their off- and on-target scores or to remove sgRNA with 0 or 1 mismatches. We recommend using “30” off-target cut-off. User can also remove all motifs with no designed guides or apply the filter “Select only motifs with no guides” to obtain the list of those motifs.

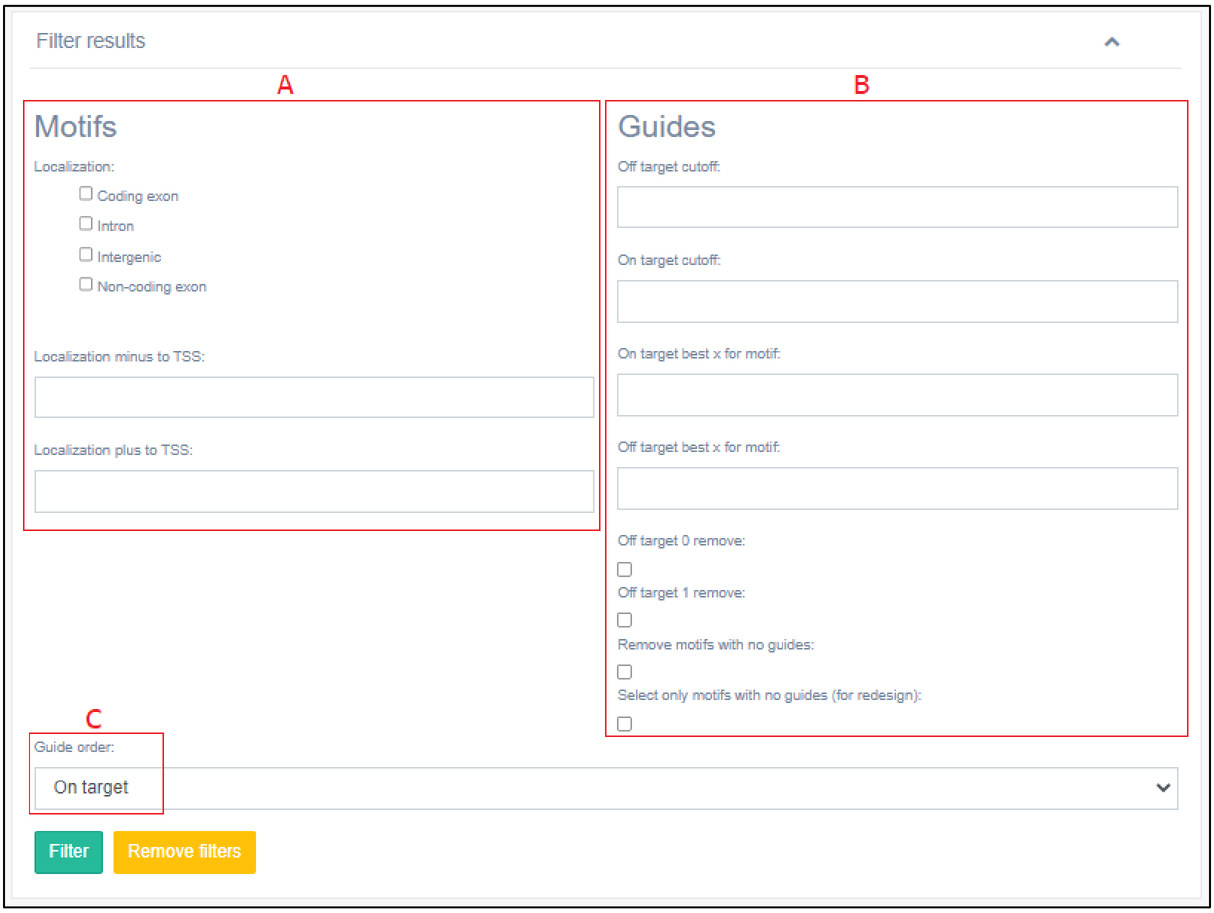

The user may also sort the order of guides [C] based on: on target score, off-target score or position within the queried sequence.

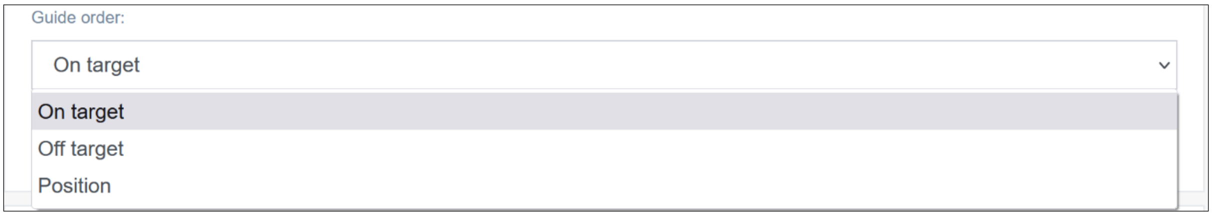

Supplement: gkad355_Supplemental_Files [file gkad355_supplemental_files.zip › transCRISPR Manual revised.pdf]
